# Design, Synthesis and Bioactivity of Novel Glycosylthiadiazole Derivatives

**DOI:** 10.3390/molecules19067832

**Published:** 2014-06-11

**Authors:** Guanghui Zong, Hanqing Zhao, Rui Jiang, Jianjun Zhang, Xiaomei Liang, Baoju Li, Yanxia Shi, Daoquan Wang

**Affiliations:** 1 Department of Applied Chemistry, China Agricultural University, Beijing 100193, China; 2 Department of Fundamental Science, Beijing University of Agriculture, Beijing 102206, China; 3 Institute of Vegetables and Flowers, Chinese Academy of Agricultural Sciences, Beijing 100081, China

**Keywords:** 1,3,4-thiadiazole, carbohydrates, synthesis, fungicidal activity

## Abstract

**A** series of novel glycosylthiadiazole derivatives, namely 2-phenylamino-5-glycosyl-1,3,4-thiadiazoles, were designed and synthesized by condensation between sugar aldehydes **A**/**B** and substituted thiosemicarbazide **C** followed by oxidative cyclization by treating with manganese dioxide. The original fungicidal activities results showed that some title compounds exhibited excellent fungicidal activities against *Sclerotinia sclerotiorum* (Lib.) de Bary and *Pyricularia oryzae* Cav, especially compounds **F-5** and **G-8** which displayed better fungicidal activities than the commercial fungicide chlorothalonil. At the same time, the preliminary studies based on the Elson-Morgan method indicated that many compounds exhibited some inhibitory activity toward glucosamine-6-phosphate synthase (GlmS). The structure-activity relationships (SAR) are discussed in terms of the effects of the substituents on both the benzene and the sugar ring.

## 1. Introduction

Carbohydrates play an important role in the field of pesticide investigation, and many natural carbohydrate products used as pesticides have shown great vitality. Validamycin [1], validoxylamine A [2], trehazolin [3], streptomycin [4] and kasugamycin [5] have already proven excellent activities against pests and fungi, and most of them are considered to be non-toxic for mammals and have no adverse effects on non-target organisms or on the environment. Many natural products containing glycosyl residues in their structures, such as the derivatives of avermecin and spinosad, are well known green pesticides and widely used in the control of many kinds of pests, taking the advantage of excellent bioactivities, good environment compatibility and structure variability [6,7,8,9]. Encouraged by the successes of the developed commercial pesticides based on natural carbohydrates, pesticide chemists have paid considerable attention to the design, synthesis and activity evaluation of novel carbohydrate-containing compounds as potential pesticides, finding that heterocyclic compounds modified by carbohydrates exhibit excellent biological activities [10,11,12,13].

It is well established that 1,3,4-thiadiazole and their derivatives exhibit a broad spectrum of biological activities not only in research on drugs with anticancer [14], antimicrobial [15], antituberculosis [16], anticonvulsant [17], or anti-inflammatory activities [18,19] but also in pesticide research such as antifungal [20,21], insecticidal [22], herbicidal [23] and also plant growth regulating agents [24]. Consequently, studies on the synthesis and bioassays of 1,3,4-thiadiazole derivatives have attracted increasing attention in the field of pesticide discovery. As pesticides, many of the 1,3,4-thiadiazole derivatives showed high toxicity profiles and were taken off the market. Thus an interest in developing novel bioactive agents with low toxicities and an acceptable impact on the environment is increasing. One way to reach this goal is to modify the parent 1,3,4-thiadiazole structure.

**Figure 1:**
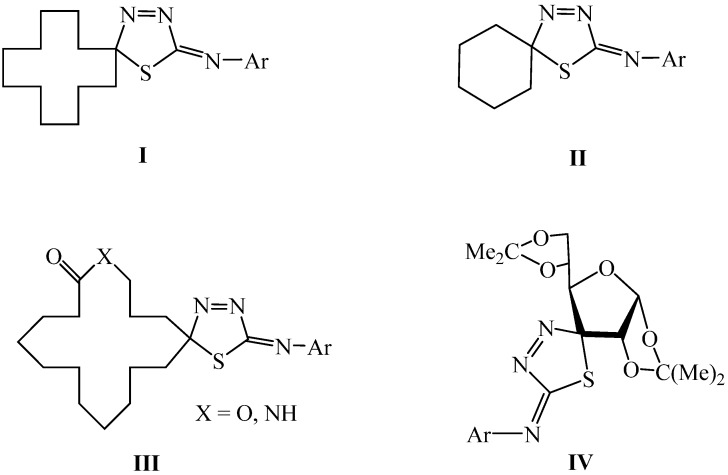
1,3,4-Thiadiazole derivatives developed in our laboratory.

In our previous studies [25,26,27], numerous 1,3,4-thiadiazole derivatives (**I-III**, Figure 1) were designed and synthesized. Some of them exhibited good fungicidal activity against *Rhizoctonia solani* Kühn, *Verticillium dahlia* Kleb. and *Pyricularia oryzae* Cav. 

**Scheme 1:**
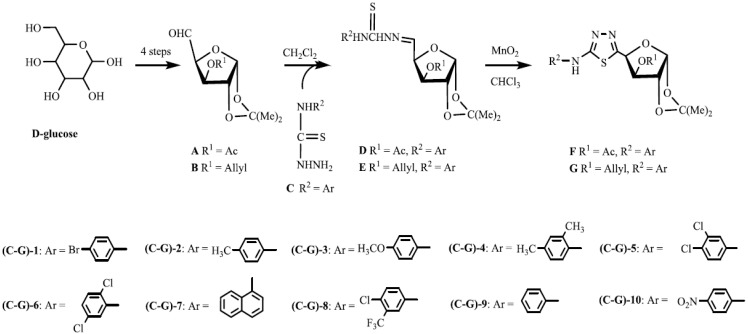
Synthesis of the target compounds **F/G**.

**Scheme 2:**
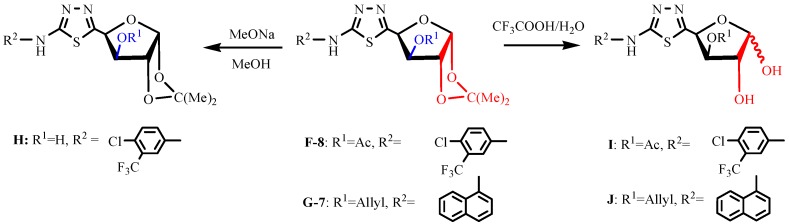
Synthesis of the sugar moiety-modified compounds.

Considering the advantages of using carbohydrates in developing novel pesticides, we made some efforts to investigate the antifungal activities of 1,3,4-thiadiazoles modified by carbohydrates, and a series of thiadiazoline derivatives containing glucofuranose were synthesized in our laboratory [28]. The fungicidal activity results obtained showed that compound **IV** (Figure 1) exhibited excellent fungicidal activities against *Phytophtora parasitica* Dast and *Helminthosporium maydis* Nisik & Miy. Inspired by these promising results, we developed a great interest in searching for potential 1,3,4-thiadiazole derivative pesticides containing furanoses. In the structure of compound **IV**, the 1,3,4-thiadiazole moiety and the glucofuranose moiety were connected in a spirocyclic manner. In particular the question of the kind of changes in the fungicidal activities that might happen if the two moieties were connected directly to each other (through a single bond) caught our attention. Thus, a series of xylose-based 1,3,4-thiadiazoles, were synthesized and evaluated, as mentioned in one of our former Chinese patents [29]. At the same time, as there is some similarity between the target compounds and the D-fructose-6-P, which is one of the substrates in the first committed step of the hexosamine biosynthesis pathway [30] by glucosamine-6-phosphate synthase (GlmS; EC 2.6.1.16), their enzyme inhibitory activities were evaluated, too. In this paper, we would like to report their synthesis (Scheme 1 and Scheme 2) and bioactivities in much greater details, and also their structure-activity relationship studies. Furthermore, to investigate the effects of the protecting groups in the sugar ring on the activities of compounds **F** and **G**, the deprotected compounds **H**, **I** and **J** were also synthesized and evaluated. We report herein the preliminary results of the study.

## 2. Results and Discussion

### 2.1. Synthesis of the Title Compounds

The synthesis of the target compounds was outlined in Scheme 1. According to the known methods [31,32,33], two furanosyl aldehydes (**A** and **B**) were prepared using D-glucose as the starting material. 

The substituted thiosemicarbazides **C** were synthesized from the corresponding substituted arylamines as previously described [24,31]. The condensation between furanosyl aldehydes **A** or **B** and substituted thiosemicarbazides **C** provided compounds **D** or **E**, respectively. Then the target compounds **F** or **G** were prepared by treating compounds **D** or **E** with MnO_2_. Compound **H** was obtained by deacetylation of compound **F-8**, and compounds **I** and **J** were obtained by deisopropylidenation of the related compounds **F-8** and **G-7**, as shown in Scheme 2.

All the derivatives were synthesized according to the procedures described in Scheme 1 and Scheme 2 in good overall yields of 65%–92%. The synthesized compounds were characterized by ^1^H-NMR, MS and HRMS. Most of the ^1^H-NMR experiments of compounds **F** and **G** were conducted in CDCl_3_ as the solvent. Nevertheless, the signal of N*H* was too weak in some cases, so we had to switch the solvent to DMSO-*d*_6_. The physical data of the target compounds are given in Table 1.

**Table 1:** Physical Data of Compounds **F/G**.

Compd.	R1	R2	Formula	Status	m.p./°C	Yield (%)
**F-1**	Ac	4-Br-C_6_H_4_-	C_17_H_18_BrN_3_O_5_S	White foamy solid	204.1–204.7	79
**F-2**	Ac	4-CH_3_-C_6_H_4_-	C_18_H_21_N_3_O_5_S	White foamy solid	187.9–188.1	82
**F-3**	Ac	4-CH_3_O-C_6_H_4_-	C_18_H_21_N_3_O_6_S	yellow foamy solid	177.7–178.7	73
**F-4**	Ac	2,4-(CH_3_)_2_-C_6_H_4_-	C_19_H_23_N_3_O_5_S	White foamy solid	133.7–134.3	81
**F-5**	Ac	3,4-Cl_2_-C_6_H_4_-	C_17_H_17_Cl_2_N_3_O_5_S	White foamy solid	147.7–148.5	83
**F-6**	Ac	2,5-Cl_2_-C_6_H_4_-	C_17_H_17_Cl_2_N_3_O_5_S	White foamy solid	61.8–62.9	85
**F-7**	Ac	1-Naphthyl-	C_21_H_21_N_3_O_5_S	White foamy solid	57.0–58.1	65
**F-8**	Ac	4-Cl-3-CF_3_-C_6_H_3_-	C_18_H_17_ClF_3_N_3_O_5_S	White foamy solid	140.0–141.2	77
**F-9**	Ac	C_6_H_4_-	C_17_H_19_N_3_O_5_S	White foamy solid	195.1–195.5	78
**F-10**	Ac	4-NO_2_-C_6_H_4_-	C_17_H_18_N_4_O_7_S	yellow foamy solid	198.4–198.7	73
**G-1**	All	4-Br-C_6_H_4_-	C_18_H_20_BrN_3_O_4_S	White foamy solid	204.1–204.7	68
**G-2**	All	4-CH_3_-C_6_H_4_-	C_19_H_23_N_3_O_4_S	White foamy solid	187.9–188.1	74
**G-3**	All	4-CH_3_O-C_6_H_4_-	C_19_H_23_N_3_O_5_S	White foamy solid	177.7–178.7	78
**G-4**	All	2,4-(CH_3_)_2_-C_6_H_4_-	C_20_H_25_N_3_O_4_S	White foamy solid	133.7–134.3	76
**G-5**	All	3,4-Cl_2_-C_6_H_4_-	C_18_H_19_Cl_2_N_3_O_4_S	White foamy solid	147.7–148.5	81
**G-6**	All	2,5-Cl_2_-C_6_H_4_-	C_18_H_19_Cl_2_N_3_O_4_S	White foamy solid	61.8–62.9	76
**G-7**	All	1-Naphthyl-	C_22_H_23_N_3_O_4_S	yellow foamy solid	57.0–58.1	68
**G-8**	All	4-Cl-3-CF_3_-C_6_H_3_-	C_19_H_19_ClF_3_N_3_O_4_S	White foamy solid	140.0–141.2	86
**G-9**	All	C_6_H_4_-	C_18_H_21_N_3_O_4_S	White foamy solid	195.1–195.5	91
**G-10**	All	4-NO_2_-C_6_H_4_-	C_18_H_20_N_4_O_6_S	White foamy solid	198.4–198.7	71

### 2.2. Fungicidal Activity of Compounds **F**/**G** against Six Fungus Species

Compounds **F/G** were evaluated in a series of *in vitro* fungicidal tests against six fungal species, and compared with the commercial fungicide chlorothalonil. As shown in Table 2, the resulting data revealed that most of the tested compounds displayed a certain degree of fungicidal activity against the six species. Among them, the majority of the compounds showed better fungicidal activity against *S. sclerotiorum* than the other five fungi. Among the 20 tested compounds, there were seven that displayed an inhibition rate of 90% or more against *S. sclerotiorum* at a concentration of 50 µg/mL. Therefore, the further activity evaluation of the compounds in our research was performed against *S. sclerotiorum*.

**Table 2:** Fungicidal activity of compounds **F**/**G** against six fungus species (% control at 50 µg/mL).

Compds No.	*S. sclerotiorum*	*P.* *parasitica Dast*	*B. cinerea*	*R. solani*	*P. oryzae Cav.*	*P.* *asparagi saecrdo*
**F-1**	68	11	14	60	29	−8
**F-2**	76	40	50	65	52	35
**F-3**	80	22	26	70	27	5
**F-4**	84	35	32	69	58	50
**F-5**	91	21	24	67	76	23
**F-6**	94	97	66	77	72	25
**F-7**	88	67	74	62	70	43
**F-8**	89	37	51	53	46	21
**F-9**	68	27	−11	63	56	54
**F-10**	52	12	6	59	41	25
**G-1**	79	30	72	58	80	26
**G-2**	87	32	79	77	81	15
**G-3**	74	65	50	80	79	66
**G-4**	93	61	88	62	87	51
**G-5**	85	74	84	56	85	84
**G-6**	90	63	77	63	87	98
**G-7**	92	42	85	68	85	66
**G-8**	98	64	80	59	83	71
**G-9**	86	40	64	55	79	85
**G-10**	91	43	81	55	78	57
Chlorothalonil	93	92	84	100	82	94

### 2.3. Precise Fungicidal Activity of Compounds **F**/**G** Against S. sclerotiorum

The precise fungicidal activity of compounds **F/G** against *S. sclerotiorum* was evaluated, and the data are shown in Table 3. For half of the compounds **F/G**, *i.e*., 10 out of the 20 tested compounds, their EC_50_ values were <3 μg/mL. They thus exhibited good fungicidal activity against *S. sclerotiorum.* Among them, compounds **F-5**, **F-8**, **G-5**, **G-6** and **G-8** (the EC_50_ values of which are 0.29, 1.50, 1.50, 1.62 and 0.46 μg/mL, respectively) exhibited excellent fungicidal activity and they are comparable with the commercial fungicide chlorothalonil (with a EC_50_ value of 0.59 μg/mL).

**Table 3:** Fungicidal Activity of Compounds **F**/**G** against *S. sclerotiorum*.

Compds No.	Regression eq	r	EC_50_ (μg/mL)	EC_90_ (μg/mL)
**F-1**	Y = 4.93 + 0.57x	0.9416	1.33	247.14
**F-2**	Y = 3.96 + 1.39x	0.9880	5.64	47.03
**F-3**	Y = 4.77 + 0.58x	0.9791	2.45	382.78
**F-4**	Y = 4.11 + 1.04x	0.9717	7.11	120.46
**F-5**	Y = 5.31 + 0.58x	0.9349	0.29	47.38
**F-6**	Y = 3.93 + 1.80x	0.9758	3.94	20.30
**F-7**	Y = 2.04 + 2.18x	0.9937	22.53	87.03
**F-8**	Y = 4.80 + 1.11x	0.9543	1.50	21.6
**F-9**	Y = 4.51 + 0.72x	0.9798	4.70	276.27
**F-10**	Y = 4.13 + 0.56x	0.9765	37.13	7558.45
**G-1**	Y = 3.84 + 1.29x	0.9059	8.01	79.59
**G-2**	Y = 3.94 + 1.58x	0.9993	4.70	30.32
**G-3**	Y = 4.75 + 0.63x	0.9457	2.51	273.14
**G-4**	Y = 2.48 + 2.52x	0.9859	9.92	31.89
**G-5**	Y = 4.83 + 0.96x	0.9880	1.50	32.10
**G-6**	Y = 4.80 + 0.94x	0.9871	1.62	37.28
**G-7**	Y = 4.68 + 1.06x	0.9940	2.00	32.30
**G-8**	Y = 5.34 + 0.99x	0.9961	0.46	8.91
**G-9**	Y = 4.32 + 1.26x	0.9809	3.44	35.81
**G-10**	Y = 4.69 + 1.00x	0.9534	2.02	38.63
Chlorothalonil	5.19 + 0.84x	0.9784	0.59	19.56

In general, the following structure-activity relationships (SAR) in compounds **F/G** were observed: (1) for the two series **F** and **G**, on an overall level the latter (R^2^ = allyl) displayed a better fungicidal activity than the former (R^2^ = Ac), *i.e.*, there were six compounds in series **G** that exhibited better fungicidal activity than their counterparts in series **F**; (2) for the **F** series, the fungicidal activity is increased by improving the electron-withdrawing ability of substituents on the benzene ring, *i.e*., in compounds **F-4** (R^2^ = 2,4-(CH_3_)_2_-C_6_H_3_-), **F-2** (R^2^ = 4-CH_3_-C_6_H_3_-), **F-9 ** (R^2^ = C_6_H_3_-), **F-3** (R^2^ = 4-CH_3_O-C_6_H_3_-) and **F-1** (R^2^ = 4-Br-C_6_H_3_-) with the EC_50_ values of 7.11, 5.64, 4.70, 2.45 and 1.33 μg/mL, respectively; (3) for the **G** series, the fungicidal activity is increased by improving the electron-withdrawing ability of substituents on the benzene ring, too, *i.e*., compounds **G-4** (R^2^ = 2,4-(CH_3_)_2_-C_6_H_3_-), **G-2** (R^2^ = 4-CH_3_-C_6_H_3_-), **G-9** (R^2^ = C_6_H_3_-), **G-3** (R^2^ = 4-CH_3_O-C_6_H_3_-) and **G-6** (R^2^ = 2,5-Cl_2_-C_6_H_3_-) with the EC_50_ values of 9.92, 4.70, 3.44, 2.51 and 1.62 μg/mL, respectively; (4) In both series **F** and **G**, the compounds with two electron-withdrawing groups in the benzene ring have the best fungicidal activity in their own series, *i.e*., compounds **F-5** (R^2^ = 3,4-Cl_2_-C_6_H_3_-) and **G-8** (R^2^ = 4-Cl-3-CF_3_-C_6_H_3_-) with the EC_50_ values of 0.29 and 0.46 μg/mL, respectively. They were slightly better than the commercial fungicide chlorothalonil (EC_50_ value = 0.59 μg/mL). Similarly, compounds **F-8** (R^2^ = 4-Cl-3-CF_3_-C_6_H_3_-) and **G-5** (R^2^ = 3,4-Cl_2_-C_6_H_3_-), both with EC_50_ values of 1.50 μg/mL, also displayed excellent fungicidal activities. However, the compounds with two electron-donating groups in the benzene ring displayed only moderate fungicidal activity, *i.e.*, compounds **F-4** (R^2^ = 2,4-Me_2_-C_6_H_3_-) and **G-4** (R_2_ = 2,4-Me_2_-C_6_H_3_-) with the EC_50_ values of 7.11 and 9.92 μg/mL, respectively.

### 2.4. Effect of Structure Modifications on the Sugar Ring around Compound **F/G**

Having identified the relatively potent compounds of series **F/G**, such as **F-8**, **G-8** and **G-7**, we next focused our attention on investigating the effects of subtle structural changes in the sugar ring of compounds **F/G**. To this end, compounds **H**, **I** and **J** were prepared and evaluated. The fungicidal results against *S. sclerotiorum* are provided in Table 4. It is evident from the data (Table 3 and Table 4) that, among all the derivatives studied above, compounds with complete OH-protection in the sugar ring, *i.e.*, compounds **F-8**, **G-8** and **G-7**, displayed the most promising results, with the EC_50_ values of 1.50, 0.46, 2.00 μg/mL respectively. Meanwhile, compound **H** without the OH-protection at the 3-position in the sugar ring (and with an EC_50_ value of 4.61 μg/mL) has shown a slightly decreased fungicidal activity. Compounds **I** and **J** without the OH-protection at both 1 and 2-position displayed a significant decrease in their fungicidal activity, with EC_50_ values of 8.18, 18.91 μg/mL, respectively. The results above demonstrate that appropriate protections of the hydroxyl groups in the sugar ring can make positive contributions to the fungicidal activity against *S. Sclerotiorum*. Interestingly, however, the enzyme inhibitory activity of compounds **H**, **I** and **J** is superior to that of compound **G**-**8** (Table 5), which may be associated with a better structural similarity between fructose 6-phosphate (Fru-6-P) and compounds **H**, **I** and **J**.

**Table 4:** Effects of structural modifications in sugar ring of compounds **F**/**G** on the activity against *S. sclerotiorum*. 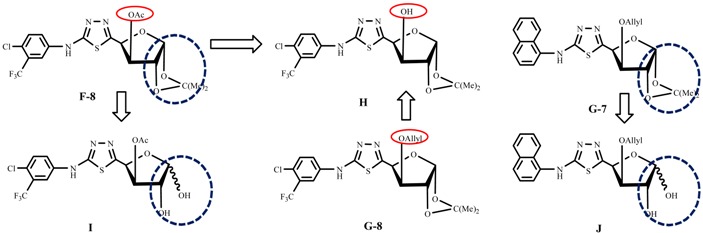

Compds No.	Regression eq	r	EC_50_	EC_90_
**H**	Y = 4.40 + 0.91x	0.8678	4.61	119.37
**I**	Y = 4.27 + 0.80x	0.9963	8.18	321.93
**J**	Y = 2.80 + 1.72x	0.9228	18.91	105.14

**Table 5:** Enzyme inhibition Rate of Compounds **F** and **G** at 0.35 mm.

Compd No.	Inhibition Rate (%)	Compd No.	Inhibition Rate (%)
**F-1**	13.2	**G-1**	12.0
**F-2**	15.3	**G-2**	10.8
**F-3**	17.7	**G-3**	16.1
**F-4**	18.5	**G-4**	15.2
**F-5**	18.3	**G-5**	25.8
**F-6**	19.1	**G-6**	17.4
**F-7**	17.9	**G-7**	28.1
**F-8**	18.1	**G-8**	26.5
**F-9**	14.7	**G-9**	20.3
**F-10**	13.3	**G-10**	24.2
**H**	29.4	**J**	36.2
**I**	35.8		

### 2.5. Bioassay of Enzyme Inhibitory Activities [34,35,36,37]

Inhibitory activity of all the synthesized compounds towards *Candida albicans* GlcN-6-P synthase was evaluated using the optimized Elson-Morgan method [38]. The absorption value of the solution was measured at 585 nm, and then the concentration was counted by the specification curve which was determined thanks to the relation between the absorption value and the concentration of glucosamine-6-phosphate. Finally the enzyme inhibition rate was calculated according to Equation (1):

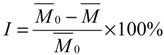
(1)


In Equation (1): *I* is the inhibition rate, 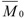 is the average concentration of glucosamine-6-phosphate in the blank test, and 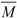 is the average concentration of glucosamine-6-phosphate in the presence of target compounds. The inhibition rates were given in Table 5 at 0.35 mm.

The compounds of series **F** and **G** exhibited some enzyme inhibitory activities (Table 5). Compounds **G**-**5**, **G**-**7**, **G**-**8** and **G**-**10** are more active against glucosamine-6-phosphate synthase than the other compounds. On the whole, the enzyme inhibitory activity of **G** series of compounds is superior to the **F** series. The enzyme inhibitory activity of compounds **H**, **I** and **J** is superior to that of compound **G**-**8** (Table 5), which may be associated with a better structural similarity between fructose 6-phosphate (Fru-6-P) and compounds **H**, **I** and **J**.

## 3. Experimental

### 3.1. General Methods

All starting materials and reagents were commercially available and used without further purification except as indicated. ^1^H-NMR (300 MHz) and ^13^C-NMR (75 MHz) spectra was recorded in CDCl_3_ or DMSO-*d*_6_ with a Bruker DPX300 spectrometer, using TMS as internal standard; Mass spectra were obtained with Agilent 1100 series LC/MSD mass spectrometer. High-resolution mass spectra (HRMS) was performed by the Peking University. Melting points were measured on a Yanagimoto melting-point apparatus and are uncorrected. 

### 3.2. Chemical Synthesis

*General Procedure for the Syntheses of Title Compounds* **F**, **G**, **H**, **I** *and* **J**. Substituted aldehydes **A** and **B** were prepared from D-glucose as the starting material according to known methods [20,21,22]. Substituted thiosemicarbazides **C** were synthesized from amines as previously described [33,39].

*General Procedure for the Synthesis of Intermediate Compounds* D/E. A solution of aldehyde **A/B** (5.5 mmol) and thiosemicarbazide **C** (5 mmol) in CH_2_Cl_2_ (100 mL) was heated to reflux for 6 h, at the end of which time TLC (eluent: 2:1 petroleum ether-EtOAc) indicated that the reaction was complete. The solvent was evaporated under diminished pressure at 40 °C to give a white solid, and the crude product was used for next step directly without purification.

*General Procedure for the Synthesis of Title Compounds* **F**/**G**. To a stirred solution of compound **D/E** (5.0 mmol) in CHCl_3_ (80 mL) was added MnO_2_ (10 g). The mixture was stirred for a further 1 h, at the end of which time TLC (eluent: 2:1 petroleum ether-EtOAc) indicated that the reaction was complete. After filtration, the filtrate was evaporated under reduced pressure to give a crude product, which was purified on silica gel column chromatography with 4:1 petroleum ether-EtOAc as the eluent to give the compounds **F/G**.

*2-(4-Bromophenylamino)-5-(2R,3S-O-isopropylidene-4S-O-acetyl-tetrahydrofuro-2,3,4-triol-5S)-1,3,4-thiadiazole* (**F-1**). Yield: 79%. White solid, mp 239.1–240.2 °C. ^1^H-NMR (DMSO-*d*_6_): δ 10.61 (s, 1H, N*H*), 7.62 (d, *J* = 8.9 Hz, 2H, Ar*H*), 7.52 (d, *J* = 8.9 Hz, 2H, Ar*H*), 6.07 (d, *J* = 3.7 Hz, 1H, H-1), 5.54 (d, *J* = 3.0 Hz, 1H, H-3), 5.28 (d, *J* = 3.0 Hz, 1H, H-4), 4.79 (d, *J* = 3.7 Hz, 1H, H-2), 2.02 (s, 3H, C*H*_3_CO), 1.52, 1.31 (2s, 6H, *Me*_2_C); ^13^C-NMR (DMSO-*d*_6_) δ 168.82, 165.18, 154.23, 139.71, 131.74, 119.36, 113.27, 111.85, 104.36, 82.63, 76.51, 76.30, 26.33, 25.93, 20.61. ESI-MS *m/z* calcd. for C_17_H_17_BrN_3_O_5_S (M-H) 454.0. Found: 454.0. HRMS for C_17_H_19_BrN_3_O_5_S [M+H]^+^ 456.0223. Found: 456.0212.

*2-(4-Tolylamino)-5-(2R,3S-O-isopropylidene-4S-O-acetyl-tetrahydrofuro-2,3,4-triol-5S)-1,3,4-thiadiazole* (**F-2**). Yield: 82%. Pale-yellow solid, mp 227.6–228.0 °C. ^1^H-NMR (CDCl_3_): δ 10.42 (br, 1H, N*H*), 7.33-7.27 (m, 2H, Ar*H*), 7.19 (m, 2H, Ar*H*), 6.03 (d, *J* = 3.6 Hz, 1H, H-1), 5.72 (d, *J* = 3.1 Hz, 1H, H-3), 5.48 (d, *J* = 3.1 Hz, 1H, H-4), 4.65 (d, *J* = 3.7 Hz, 1H, H-2), 2.34 (s, 3H Ar-C*H*_3_), 2.00 (s, 3H, C*H*_3_CO), 1.59, 1.36 (2s, 6H, *Me*_2_C); ^13^C-NMR (CDCl_3_) δ 169.01, 138.05, 133.25, 130.13, 118.45, 112.86, 104.77, 83.30, 77.26, 76.92, 26.75, 26.22, 20.79. ESI-MS *m/z* calcd. for C_18_H_21_N_3_NaO_5_S (M+Na) 414.1. Found: 414.2. HRMS for C_18_H_22_N_3_O_5_S [M+H]^+^ 392.1275. Found: 392.1275.

*2-(4-Methoxyphenylamino)-5-(2R,3S-O-isopropylidene-4S-O-acetyl-tetrahydrofuro-2,3,4-triol-5S)-1,3,4-thiadiazols* (**F-3**). Yield: 73%. Yellow solid, mp 186.7–188.0 °C. ^1^H-NMR (DMSO-*d*_6_): δ 10.28 (br, 1H, N*H*), 7.52 (d, *J* = 8.9 Hz, 2H, Ar*H*), 6.93 (d, *J* = 8.9 Hz, 2H, Ar*H*), 6.05 (d, *J* = 3.8 Hz, 1H, H-1), 5.50 (d, *J* = 3.0 Hz, 1H, H-3), 5.26 (d, *J* = 3.1 Hz, 1H, H-4), 4.76 (d, *J* = 3.8 Hz, 1H, H-2), 3.73 (s, 3H, Ar-C*H*_3_O), 2.02 (s, 3H, C*H*_3_CO), 1.51, 1.30 (2s, 6H, *Me*_2_C); ^13^C-NMR (CDCl_3_) δ 169.08, 156.67, 133.90, 121.40, 114.97, 112.97, 104.83, 83.34, 77.29, 77.00, 55.61, 26.81, 26.29, 20.88. ESI-MS *m/z* calcd. for C_18_H_22_N_3_O_6_S [M+H] 408.1. Found: 408.1. HRMS for C_18_H_22_N_3_O_6_S [M+H]^+^ 408.1224. Found: 408.1218.

*2-(2,4-Dimethylphenylamino)-5-(2R,3S-O-isopropylidene-4S-O-acetyl-tetrahydrofuro-2,3,4-triol-5S)-1,3,4-thiadiazols* (**F-4**). Yield: 81%. White solid, mp 155.9–156.1 °C. ^1^H-NMR (CDCl_3_): δ 7.79 (br, 1H, N*H*), 7.29 (d, *J* = 8.0 Hz, 1H, Ar*H*), 7.10–7.00 (m, 2H, Ar*H*), 5.97 (d, *J* = 3.6 Hz, 1H, H-1), 5.65 (d, *J* = 3.1 Hz, 1H, H-3), 5.43 (d, *J* = 3.1 Hz, 1H, H-4), 4.61 (d, *J* = 3.7 Hz, 1H, H-2), 2.32 (2s, 6H, Ar-C*H*_3_), 1.98 (s, 3H, C*H*_3_CO), 1.56, 1.34 (2s, 6H, *Me*_2_C); ^13^C-NMR (CDCl_3_) δ 172.26, 168.97, 152.98, 137.24, 136.09, 132.52, 132.10, 127.69, 123.01, 112.83, 104.71, 83.26, 77.28, 76.93, 26.75, 26.25, 20.93, 20.78, 17.89. ESI-MS *m/z* calcd. for C_19_H_24_N_3_O_5_S [M+H] 406.1. Found: 406.1. HRMS for C_19_H_24_N_3_O_5_S [M+H]^+^ 406.1431. Found: 406.1422.

*2-(3,4-Dichlorophenylamino)-5-(2R,3S-O-isopropylidene-4S-O-acetyl-tetrahydrofuro-2,3,4-triol-5S)-1,3,4-thiadiazole* (**F-5**). Yield: 83%. White solid, mp 232.6–235.0 °C. ^1^H-NMR (DMSO-*d*_6_): δ 10.79 (s, 1H, N*H*), 8.07 (d, *J* = 2.5 Hz, 1H, Ar*H*), 7.59 (d, *J* = 8.8 Hz, 1H, Ar*H*), 7.49 (dd, *J* = 8.9, 2.5 Hz, 1H, Ar*H*), 6.08 (d, *J* = 3.7 Hz, 1H, H-1), 5.56 (d, *J* = 3.1 Hz, 1H, H-3), 5.30 (d, *J* = 3.1 Hz, 1H, H-4), 4.79 (d, *J* = 3.8 Hz, 1H, H-2), 2.02 (s, 3H, C*H*_3_CO), 1.52, 1.31 (2s, 6H, *Me*_2_C); ^13^C-NMR (DMSO-*d*_6_) δ 168.81, 164.87, 154.87, 140.24, 131.30, 130.72, 123.15, 118.55, 117.53, 111.88, 104.40, 82.63, 76.47, 76.29, 26.32, 25.91, 20.59. ESI-MS *m/z* calcd. for C_17_H_18_C_l2_N_3_O_5_S [M+H] 446.0. Found: 446.1. HRMS for C_17_H_18_C_l2_N_3_O_5_S [M+H]^+^ 446.0339. Found: 446.0330.

*2-(2,5-Dichlorophenylamino)-5-(2R,3S-O-isopropylidene-4S-O-acetyl-tetrahydrofuro-2,3,4-triol-5S)-1,3,4-thiadiazole* (**F-6**). Yield: 85%. White solid, mp 119.6–120.6 °C. ^1^H-NMR (CDCl_3_): δ 8.22 (d, *J* = 2.3 Hz, 1H, Ar*H*), 7.69 (s, 1H, N*H*), 7.33 (d, *J* = 8.5 Hz, 1H, Ar*H*), 7.00 (dd, *J* = 8.5, 2.4 Hz, 1H, Ar*H*), 6.05 (d, *J* = 3.6 Hz, 1H, H-1), 5.73 (d, *J* = 3.1 Hz, 1H, H-3), 5.50 (d, *J* = 3.1 Hz, 1H, H-4), 4.68 (d, *J* = 3.6 Hz, 1H, H-3), 2.04 (s, 3H, C*H*_3_CO), 1.59, 1.36 (2s, 6H, *Me*_2_C); ^13^C-NMR (CDCl_3_) δ 169.02, 165.30, 156.89, 137.41, 133.82, 130.28, 123.74, 120.59, 119.02, 113.09, 104.92, 83.30, 77.18, 77.04, 26.80, 26.27, 20.83. ESI-MS *m/z* calcd. for C_17_H_18_C_l2_N_3_O_5_S [M+H] 446.0. Found: 446.1. HRMS for C_17_H_18_C_l2_N_3_O_5_S [M+H]^+^ 446.0339. Found: 446.0329.

*2-(Naphthalen-1-ylamino)-5-(2R,3S-O-isopropylidene-4S-O-acetyl-tetrahydrofuro-2,3,4-triol-5S)-1,3,4-thiadiazole* (**F-7**). Yield: 65%. Pale-yellow solid, mp 186.1–186.9 °C. ^1^H-NMR (DMSO-*d*_6_): δ 10.36 (s, 1H, N*H*), 8.25 (m, 1H, Ar*H*), 8.13 (d, *J* = 7.5 Hz, 1H, Ar*H*), 7.96 (m, 1H, Ar*H*), 7.72 (d, *J* = 8.2 Hz, 1H, Ar*H*), 7.64–7.50 (m, 3H, Ar*H*), 6.06 (d, *J* = 3.8 Hz, 1H, H-1), 5.54 (d, *J* = 3.1 Hz, 1H, H-3), 5.29 (d, *J* = 3.1 Hz, 1H, H-4), 4.78 (d, *J* = 3.8 Hz, 1H, H-2), 2.01 (s, 3H, C*H*_3_CO), 1.52, 1.30 (2s, 6H, *Me*_2_C); ^13^C-NMR (75 MHz, DMSO-*d*_6_) δ 168.83, 167.94, 153.96, 136.33, 133.93, 128.33, 126.21, 126.01, 125.95, 125.90, 124.06, 121.99, 117.28, 111.79, 104.30, 82.61, 76.59, 76.28, 26.32, 25.92, 20.61. ESI-MS *m/z* calcd. for C_21_H_22_N_3_O_5_S [M+H] 428.1. Found: 428.1. HRMS for C_21_H_22_N_3_O_5_S [M+H]^+^ 428.1275. Found: 428.1263.

*2-(4-Chloro-3-(trifluoromethyl)phenylamino)-5-(2R,3S-O-isopropylidene-4S-O-acetyl-tetrahydrofuro-2,3,4-triol-5S)-1,3,4-thiadiazole* (**F-8**). Yield: 77%. Pale-yellow solid, mp 219.8–221.5 °C. ^1^H-NMR (DMSO-*d_6_*): δ 10.95 (s, 1H, N*H*), 8.25 (d, *J* = 2.5 Hz, 1H, Ar*H*), 7.86 (dd, *J* = 8.8, 2.5 Hz, 1H, Ar*H*), 7.69 (d, *J* = 8.8 Hz, 1H, Ar*H*), 6.09 (d, *J* = 3.7 Hz, 1H, H-1), 5.57 (d, *J* = 3.1 Hz, 1H, H-3), 5.30 (d, *J* = 3.1 Hz, 1H, H-4), 4.80 (d, *J* = 3.7 Hz, 1H, H-2), 2.02 (s, 3H, C*H*_3_CO), 1.52, 1.31 (2s, 6H, *Me*_2_C); ^13^C-NMR (DMSO-*d*_6_) δ 168.84, 164.88, 155.12, 139.63, 132.26, 126.99 (q, *J* = 30.7 Hz), 124.53, 122.25 (d, *J* = 1.7 Hz), 122.11, 120.91, 116.03 (q, *J* = 5.5 Hz), 111.91, 104.43, 82.63, 76.39 (d, *J* = 11.6 Hz), 26.32, 25.92, 20.57. ESI-MS *m/z* calcd. for C_18_H_18_ClF_3_N_3_O_5_S [M+H] 480.1. Found: 480.1. HRMS for C_18_H_18_ClF_3_N_3_O_5_S [M+H]^+^ 480.0602. Found: 480.0588.

*2-(Phenylamino)-5-(2R,3S-O-isopropylidene-4S-O-acetyl-tetrahydrofuro-2,3,4-triol-5S)-1,3,4-thiadiazole* (**F-9**). Yield: 78%. Pale-yellow solid, mp 213.7–214.1 °C. ^1^H-NMR (DMSO-*d*_6_): δ 10.63 (s, 1H, N*H*), 7.41-7.39 (m, 4H, Ar*H*), 7.11 (m, 1H, Ar*H*), 6.04 (d, *J* = 3.6 Hz, 1H, H-1), 5.74 (d, *J* = 3.1 Hz, 1H, H-3), 5.51 (d, *J* = 3.1 Hz, 1H, H-4), 4.66 (d, *J* = 3.7 Hz, 1H, H-2), 2.00 (s, 3H, C*H*_3_CO), 1.60, 1.36 (2s, 6H, *Me*_2_C); ^13^C-NMR (DMSO-*d*_6_) δ 168.84, 165.55, 153.68, 140.43, 129.05, 121.97, 117.43, 111.82, 104.33, 82.62, 76.54, 76.30, 26.32, 25.93, 20.61. ESI-MS *m/z* calcd. for C_17_H_20_N_3_O_5_S [M+H] 378.1. Found: 378.1. HRMS for C_17_H_20_N_3_O_5_S [M+H]^+^ 378.1118. Found: 378.1109.

*2-(4-Nitrophenylamino)-5-(2R,3S-O-isopropylidene-4S-O-acetyl-tetrahydrofuro-2,3,4-triol-5S)-1,3,4-thiadiazole* (**F-10**). Yield: 73%. Pale-yellow solid, mp 233.9–235.5 °C. ^1^H-NMR (DMSO-*d*_6_): δ 11.22 (s, 1H, N*H*), 8.27 (d, *J* = 8.7 Hz, 2H, Ar*H*), 7.85 (d, *J* = 8.7 Hz, 2H, Ar*H*), 6.10 (d, *J* = 3.4 Hz, 1H, H-1), 5.59 (d, *J* = 2.6 Hz, 1H, H-3), 5.32 (d, *J* = 2.6 Hz, 1H, H-4), 4.80 (d, *J* = 3.5 Hz, 1H, H-2), 2.02 (s, 3H, C*H*_3_CO), 1.53, 1.31 (2s, 6H, *Me*_2_C); ^13^C-NMR (DMSO-*d*_6_) δ 168.62, 164.27, 155.82, 145.85, 140.67, 125.20, 116.75, 111.69, 104.22, 82.40, 76.22, 76.07, 26.10, 25.71, 20.36. ESI-MS *m/z* calcd. for C_17_H_17_N_4_O_7_S (M-H) 421.1. Found: 421.0. HRMS for C_17_H_19_N_4_O_7_S [M+H]^+^ 423.0969. Found: 423.0957.

*2-(4-Bromophenylamino)-5-(2R,3S-O-isopropylidene-4S-O-allyl-tetrahydrofuro-2,3,4-triol-5S)-1,3,4-thiadiazol* (**G-1**). Yield: 68%. Pale-yellow solid, mp 204.1–204.7 °C. ^1^H-NMR (CDCl_3_): δ 10.50 (br, 1H, N*H*), 7.49-7.46 (m, 2H, Ar*H*), 7.36-7.26 m, 2H, Ar*H*), 6.04 (d, *J* = 3.6 Hz, 1H, H-1), 5.72 (m, 1H, CH_2_=C*H*CH_2_), 5.63 (d, *J* = 3.1 Hz, 1H, H-3), 5.23-5.14 (m, 2H, C*H*_2_=CHCH_2_), 4.69 (d, *J* = 3.7 Hz, 1H, H-2), 4.15 (d, *J* = 3.2 Hz, 1H, H-4), 4.04-3.86 (m, 2H, CH_2_=CHC*H*_2_), 1.58, 1.37 (2s, 6H, *Me*_2_C); ^13^C-NMR (DMSO-*d*_6_) δ 165.56, 155.14, 139.84, 134.03, 131.70, 119.28, 117.15, 113.08, 111.47, 111.39, 104.44, 81.82, 81.78, 77.57, 70.44, 26.52, 25.96. ESI-MS *m/z* calcd. for C_18_H_19_BrN_3_O_4_S (M-H) 452.0. Found: 451.9. HRMS for C_18_H_21_BrN_3_O_4_S [M+H]^+^ 454.0431. Found: 454.0415.

*2-(4-Tolylamino)-5-(2R,3S-O-isopropylidene-4S-O-allyl-tetrahydrofuro-2,3,4-triol-5S)-1,3,4-thiadiazole* (**G-2**). Yield: 74%. Pale-yellow solid, mp 187.9–188.1 °C. ^1^H-NMR (CDCl_3_): δ 9.39 (br, 1H, N*H*), 7.29–7.26 (m, 2H, Ar*H*), 7.18-7.16 (m, 2H, Ar*H*), 6.02 (d, *J* = 3.7 Hz, 1H, H-1), 5.73 (m, 1H, CH_2_=C*H*CH_2_), 5.62 (d, *J* = 3.1 Hz, 1H, H-3), 5.23-5.13 (m, 2H, C*H*_2_=CHCH_2_), 4.67 (d, *J* = 3.6 Hz, 1H, H-2), 4.14 (d, *J* = 3.1 Hz, 1H, H-4), 4.02–3.85 (m, 2H, CH_2_=CHC*H*_2_), 2.33 (s, 3H, Ar-C*H*_3_), 1.56, 1.36 (2s, 6H, *Me*_2_C); ^13^C-NMR (DMSO-*d*_6_) δ 166.13, 154.21, 138.20, 134.08, 130.77, 129.40, 117.46, 117.11, 111.34, 104.40, 81.81, 77.62, 70.42, 26.53, 25.96, 20.28. ESI-MS *m/z* calcd. for C_19_H_24_N_3_O_4_S [M+H] 390.1. Found: 390.1. HRMS for C_19_H_24_N_3_O_4_S [M+H]^+^ 390.1482. Found: 390.1468.

*2-(4-Methoxyphenylamino)-5-(2R,3S-O-isopropylidene-4S-O-allyl-tetrahydrofuro-2,3,4-triol-5S)-1,3,4-thiadiazole* (**G-3**). Yield: 78%. Pale-yellow solid, mp 177.7–178.7 °C. ^1^H-NMR (CDCl_3_): δ 9.70 (br, 1H, N*H*), 7.35–7.26 (m, 2H, Ar*H*), 6.93–6.90 (m, 2H, Ar*H*), 6.01 (d, *J* = 3.5 Hz, 1H, H-1), 5.72 (m, 1H, CH_2_=C*H*CH_2_), 5.59 (d, *J* = 3.0 Hz, 1H, H-3), 5.22–5.13 (m, 2H, C*H*_2_=CHCH_2_), 4.66 (d, *J* = 3.6 Hz, 1H, H-2), 4.13 (d, *J* = 3.0 Hz, 1H, H-4), 4.01–3.85 (m, 2H, CH_2_=CHC*H*_2_), 3.81 (s, 3H, Ar-C*H*_3_O), 1.56, 1.35 (2s, 6H, *Me*_2_C); ^13^C-NMR (DMSO-*d*_6_) δ 166.58, 154.53, 153.85, 134.12, 134.08, 119.21, 117.11, 114.26, 111.35, 104.39, 81.83, 77.65, 70.43, 55.19, 26.52, 25.96. ESI-MS *m/z* calcd. for C_19_H_24_N_3_O_5_S [M+H] 406.1. Found: 406.2. HRMS for C_19_H_24_N_3_O_5_S [M+H]^+^ 406.1431. Found: 406.1417.

*2-(2,4-Dimethylphenylamino)-5-(2R,3S-O-isopropylidene-4S-O-allyl-tetrahydrofuro-2,3,4-triol-5S)-1,3,4-thiadiazole* (**G-4**). Yield: 76%. Pale-yellow solid, mp 133.7–134.3 °C. ^1^H-NMR (CDCl_3_): δ 9.41 (s, 1H, N*H*), 7.58 (d, *J* = 9.0 Hz, 1H, Ar*H*), 7.04–6.99 (m, 2H, Ar*H*), 5.96 (d, *J* = 3.7 Hz, 1H, H-1), 5.82 (m, 1H, CH_2_=C*H*CH_2_), 5.53 (d, *J* = 3.1 Hz, 1H, H-3), 5.22–5.10 (m, 2H, C*H*_2_=CHCH_2_), 4.79 (d, *J* = 3.7 Hz, 1H, H-2), 4.07 (d, *J* = 3.1 Hz, 1H, H-4), 4.12–3.87 (m, 2H, CH_2_=CHC*H*_2_), 2.25, 2.21 (2s, 6H, Ar-C*H*_3_), 1.46,1.29 (2s, 6H, *Me*_2_C); ^13^C-NMR (DMSO-*d*_6_) δ 168.89, 154.03, 136.75, 134.08, 133.48, 131.25, 129.92, 127.02, 122.20, 117.03, 111.29, 104.33, 81.83, 77.67, 70.40, 26.51, 25.95, 20.34, 17.72. ESI-MS *m/z* calcd. for C_20_H_26_N_3_O_4_S [M+H] 404.1. Found: 404.1. HRMS for C_20_H_26_N_3_O_4_S [M+H]^+^ 404.1639. Found: 404.1624.

*2-(3,4-Dichlorophenylamino)-5-(2R,3S-O-isopropylidene-4S-O-allyl-tetrahydrofuro-2,3,4-triol-5S)-1,3,4-thiadiazole* (**G-5**). Yield: 81%. Pale-yellow solid, mp 147.7–148.5 °C. ^1^H-NMR (CDCl_3_): δ 10.77 (s, 1H, N*H*), 7.59 (s, 1H, Ar*H*), 7.44 (m, 1H, Ar*H*), 7.34 (m, 1H, Ar*H*), 6.06 (d, *J* = 3.5 Hz, 1H, H-1), 5.74 (m, 1H, CH_2_=C*H*CH_2_), 5.65 (s, 1H, H-3), 5.24–5.15 (m, 2H, C*H*_2_=CHCH_2_), 4.71 (d, *J* = 3.5 Hz, 1H, H-2), 4.42–3.82 (m, 3H, H-4, CH_2_=CHC*H*_2_), 1.59, 1.38 (2s, 6H, *Me*_2_C); ^13^C-NMR (DMSO-*d*_6_) δ 165.25, 155.74, 140.37, 134.01, 131.25, 130.73, 122.95, 118.44, 117.48, 117.16, 111.41, 104.47, 81.79, 81.75, 77.50, 70.42, 26.51, 25.94. ESI-MS *m/z* calcd. for C_18_H_20_C_l2_N_3_O_4_S [M+H] 444.0. Found: 444.0. HRMS for C_18_H_20_C_l2_N_3_O_4_S [M+H]^+^ 444.0546. Found: 444.0526.

*2-(2,5-Dichlorophenylamino)-5-(2R,3S-O-isopropylidene-4S-O-allyl-tetrahydrofuro-2,3,4-triol-5S)-1,3,4-thiadiazole* (**G-6**). Yield: 76%. Pale-yellow solid, mp 61.8–62.9 °C. ^1^H-NMR (CDCl_3_): δ 8.21 (m, 1H, Ar*H*), 7.73 (br, 1H, N*H*), 7.30 (m, 1H, Ar*H*), 6.98 (m, 1H, Ar*H*), 6.05 (d, *J* = 3.6 Hz, 1H, H-1), 5.74 (m, 1H, CH_2_=C*H*CH_2_), 5.63 (d, *J* = 3.2 Hz, 1H, H-3), 5.25–5.16 (m, 2H, C*H*_2_=CHCH_2_), 4.70 (d, *J* = 3.6 Hz, 1H, H-2), 4.18 (d, *J* = 3.2 Hz, 1H, H-4), 4.05–3.87 (m, 2H, CH_2_=CHC*H*_2_), 1.57, 1.37 (2s, 6H, *Me*_2_C); ^13^C-NMR (DMSO-*d*_6_) δ 165.69, 157.21, 138.18, 134.05, 132.07, 130.79, 122.92, 120.35, 119.94, 117.16, 111.42, 104.50, 81.83, 77.66, 70.45, 26.52, 25.96. ESI-MS *m/z* calcd. for C_18_H_20_C_l2_N_3_O_4_S [M+H] 444.0. Found: 444.0. HRMS for C_18_H_20_C_l2_N_3_O_4_S [M+H]^+^ 444.0546. Found: 444.0527.

*2-(Naphthalen-1-ylamino)-5-(2R,3S-O-isopropylidene-4S-O-allyl-tetrahydrofuro-2,3,4-triol-5S)-1,3,4-thiadiazole* (**G-7**). Yield: 68%. Pale-yellow solid, mp 57.0–58.1 °C. ^1^H-NMR (CDCl_3_): δ 10.28 (br, 1H, N*H*), 8.23 (m, 1H, Ar*H*), 8.12 (m, 1H, Ar*H*), 7.95 (m, 1H, Ar*H*), 7.00 (m, 1H, Ar*H*), 7.58-7.49 (m, 3H, Ar*H*), 5.98 (d, *J* = 3.6 Hz, 1H, H-1), 5.78 (m, 1H, CH_2_=C*H*CH_2_), 5.37 (d, *J* = 3.1 Hz, 1H, H-3), 5.22–5.10 (m, 2H, C*H*_2_=CHCH_2_), 4.81 (d, *J* = 3.7 Hz, 1H, H-2), 4.14–3.64 (m, 3H, H-4, CH_2_=CHC*H*_2_), 1.48, 1.30 (2s, 6H, *Me*_2_C); ^13^C-NMR (DMSO-*d*_6_) δ 168.27, 154.99, 136.47, 134.05, 133.94, 128.30, 126.18, 126.03, 125.93, 125.83, 123.88, 122.04, 117.10, 111.36, 104.42, 81.85, 77.71, 70.44, 26.53, 25.97. ESI-MS *m/z* calcd. for C_22_H_24_N_3_O_4_S [M+H] 426.1. Found: 426.1. HRMS for C_22_H_24_N_3_O_4_S [M+H]^+^ 426.1482. Found: 426.1462.

*2-(4-Chloro-3-(trifluoromethyl)phenylamino)-5-(2R,3S-O-isopropylidene-4S-O-allyl-tetrahydrofuro-2,3,4-triol-5S)-1,3,4-thiadiazole* (**G-8**). Yield: 86%. Pale-yellow solid, mp 140.0–141.2 °C. ^1^H-NMR (CDCl_3_): δ 10.54 (s, 1H, N*H*), 7.81 (m, 1H, Ar*H*), 7.63-7.48 (m, 2H, Ar*H*), 6.05 (d, *J* = 3.5 Hz, 1H, H-1), 5.74 (m, 1H, CH_2_=C*H*CH_2_), 5.64 (d, *J* = 3.1 Hz, 1H, H-3), 5.24–5.15 (m, 2H, C*H*_2_=CHCH_2_), 4.71 (d, *J* = 3.5 Hz, 1H, H-2), 4.17 (d, *J* = 3.0 Hz, 1H, H-4), 4.07–3.87 (m, 2H, CH_2_=CHC*H*_2_), 1.59, 1.38 (2s, 6H, *Me*_2_C); ^13^C-NMR (DMSO-*d*_6_) δ 165.26, 155.95, 139.76, 134.01, 132.21, 126.96 (q, *J* = 30.6 Hz), 124.53, 122.06 (d, *J* = 1.9 Hz), 122.01, 120.91, 117.13, 115.94 (q, *J* = 5.6 Hz), 111.44, 104.51, 81.81 (d, *J* = 2.1 Hz), 77.53, 70.45, 26.50, 25.93. ESI-MS *m/z* calcd. for C_19_H_20_ClF_3_N_3_O_4_S [M+H] 478.1. Found: 478.2. HRMS for C_19_H_20_ClF_3_N_3_O_4_S [M+H]^+^ 478.0810. Found: 478.0802.

*2-(Phenylamino)-5-(2R,3S-O-isopropylidene-4S-O-allyl-tetrahydrofuro-2,3,4-triol-5S)-1,3,4-thiadiazole* (**G-9**). Yield: 91%. Pale-yellow solid, mp 195.1–195.5 °C. ^1^H-NMR (CDCl_3_): δ 10.66 (s, 1H, N*H*), 7.46–7.35 (m, 4H, Ar*H*), 7.09 (m, 1H, Ar*H*), 6.04 (d, *J* = 3.6 Hz, 1H, H-1), 5.73 (m, 1H, CH_2_=C*H*CH_2_), 5.65 (d, *J* = 3.1 Hz, 1H, H-3), 5.23–5.13 (m, 2H, C*H*_2_=CHCH_2_), 4.69 (d, *J* = 3.6 Hz, 1H, H-2), 4.16 (d, *J* = 3.1 Hz, 1H, H-4), 4.13–3.86 (m, 2H, CH_2_=CHC*H*_2_), 1.58, 1.37 (2s, 6H, *Me*_2_C); ^13^C-NMR (DMSO-*d*_6_) δ 165.96, 154.62, 140.58, 134.06, 129.01, 121.80, 117.35, 117.11, 111.37, 104.43, 81.82, 77.62, 70.43, 26.52, 25.97. ESI-MS *m/z* calcd. for C_18_H_21_N_3_O_4_SNa (M+Na) 398.1. Found: 398.1. HRMS for C_18_H_22_N_3_O_4_S [M+H]^+^ 376.1326. Found: 376.1323.

*2-(4-Nitrophenylamino)-5-(2R,3S-O-isopropylidene-4S-O-allyl-tetrahydrofuro-2,3,4-triol-5S)-1,3,4-thiadiazole* (**G-10**). Yield: 71%. Pale-yellow solid, mp 198.4–198.7 °C. ^1^H-NMR (CDCl_3_): δ 11.75 (s, 1H, N*H*), 8.33–8.28 (m, 2H, Ar*H*), 7.62–7.57 (m, 2H, Ar*H*), 6.08 (d, *J* = 3.6 Hz, 1H, H-1), 5.80–5.69 (m, 2H, CH_2_=C*H*CH_2_, H-3), 5.25–5.15 (m, 2H, C*H*_2_=CHCH_2_), 4.74 (d, *J* = 3.7 Hz, 1H, H-2), 4.21 (d, *J* = 3.2 Hz, 1H, H-4), 4.16-3.89 (m, 2H, CH_2_=CHC*H*_2_), 1.62, 1.40 (2s, 6H, *Me*_2_C); ^13^C-NMR (DMSO-*d*_6_) δ 164.86, 156.82, 146.19, 140.78, 134.00, 125.32, 117.16, 116.84, 111.48, 104.55, 81.85, 81.79, 77.54, 70.49, 26.49, 25.91. ESI-MS *m/z* calcd. for C_18_H_21_N_4_O_6_S [M+H] 421.1. Found: 421.1. HRMS for C_18_H_21_N_4_O_6_S [M+H]^+^ 421.1176. Found: 421.1173.

*2-(4-Chloro-3-(trifluoromethyl)phenylamino)-5-(2R,3S-O-isopropylidene-4S-tetrahydrofuro-2,3,4-triol-5S)-1,3,4-thiadiazole* (**H**). To a solution of **F**-**8** (0.48 g, 1.0 mmol) in MeOH (20 mL) was added MeONa (0.05 g). The reaction mixture was stirred at rt for 0.5 h, at the end of which time TLC (1:2 petroleum ether–EtOAc) indicated that the reaction was complete. Neutralization of the reaction mixture with acidic ion exchange resin (Amberlite IR-120 (H^+^), Alfa Aesar, Tianjin, China) was conducted, and the organic phase was concentrated under reduced pressure to give a crude product, which could be purified by recrystallization from a mixture solvents of petroleum ether (10 mL) and EtOAc (2 mL). Yield: 92%. White solid, mp 249.8–250.3 °C. ^1^H-NMR (DMSO-*d*_6_): δ 10.84 (s, 1H, N*H*), 8.26 (d, *J* = 2.5 Hz, 1H, Ar*H*), 7.86 (dd, *J* = 2.5, 8.8 Hz, 2H, Ar*H*), 7.68 (d, *J* = 8.8 Hz, 1H, Ar*H*), 6.01 (d, 1H, *J* = 3.6 Hz, H-1), 5.98 (d, *J* = 5.2 Hz, 1H, O*H*), 5.33 (d, *J* = 2.7 Hz, 1H, H-4), 4.60 (d, *J* = 3.6 Hz, 1H, H-2), 4.68 (dd, *J* = 3.6 Hz, 5.2 Hz, 1H, H-3), 1.48, 1.29 (2s, 6H, *Me*_2_C); ^13^C-NMR (DMSO-*d*_6_) δ 165.13, 156.93, 139.88, 132.26, 127.00 (q, *J* = 30.7 Hz), 124.58, 121.97, 120.96, 115.93 (q, *J* = 5.6 Hz), 111.33, 104.52, 84.86, 78.49, 74.58, 26.66, 26.01. ESI-MS *m/z* calcd. for C_16_H_16_ClF_3_N_3_O_4_S [M+H] 438.0. Found: 438.0. HRMS for C_16_H_16_ClF_3_N_3_O_4_S [M+H]^+^ 438.0497. Found: 438.0478.

*2-(4-Chloro-3-(trifluoromethyl)phenylamino)-5-(3S,4S-O-acetyl-tetrahydrofuro-2,3,4-triol-5S)-1,3,4-thiadiazole* (**I**). A solution of **F-8** (0.48 g, 1.0 mmol) and CF_3_COOH (9 mL) and H_2_O (1 mL) was stirred at rt for 12 h, at the end of which time TLC (1:2 petroleum ether-EtOAc) indicated that the reaction was complete. The reaction mixture was neutralized with solid NaHCO_3_, and filtered through Celite. The filtrate was evaporated under reduced pressure to give a crude product, which was purified on silica gel column chromatography with 1:1 petroleum ether-EtOAc as the eluent to give the compound. Yield: 79%. Pale-yellow solid, mp 115.6–116.5 °C. ^1^H-NMR (DMSO-*d*_6_): δ 10.87 (d, *J* = 12.2 Hz, 1H, N*H*), 8.26 (d, *J* = 2.6 Hz, 1H, Ar*H*), 7.83 (dd, *J* = 2.6, 8.8 Hz, 2H, Ar*H*), 7.68 (d, *J* = 8.8 Hz, 1H, Ar*H*), 7.05 (br, 1H, O*H*), 5.89 (br, 1H, O*H*), 5.55–5.52 (m, 1H, H-1), 5.40–5.26 (m, 1H, H-3), 5.19–5.17 (m, 1H, H-4), 4.13–4.00 (m, 1H, H-2), 1.92 (s, 3H, OC*H*_3_). ESI-MS *m/z* calcd. for C_15_H_12_ClF_3_N_3_O_5_S (M-H) 438.0. Found: 438.0. HRMS for C_15_H_14_ClF_3_N_3_O_5_S [M+H]^+^ 440.0289. Found: 440.0272.

*2-(Naphthalen-1-ylamino)-5-(3S,4S-O-acetyl-tetrahydrofuro-2,3,4-triol-5S)-1,3,4-thiadiazole* (**J**). Deisopropylidenation of **G-7** (0.52 g, 1.2 mmol) was accomplished by following the same procedure employed for the preparation of compound **J**. Yield: 73%. Yellow solid, mp 57.2–59.7 °C. ^1^H-NMR (DMSO-*d*_6_): δ 10.19 (s, 1H, N*H*), 8.24–8.22 (m, 1H, Ar*H*), 8.09 (d, *J* = 7.4 Hz, 1H, Ar*H*), 7.97–7.94 (m, 1H, Ar*H*), 7.70 (d, *J* = 8.1 Hz, 1H, Ar*H*), 7.58–7.49 (m, 3H, Ar*H*), 6.62 (d, *J* = 5.5 Hz, 0.5H), 6.51 (d, *J* = 7.3 Hz, 0.5H), 5.77–5.70 (m, 1H, CH_2_=C*H*CH_2_), 5.57 (d, *J* = 4.3 Hz, 0.5H), 5.43–5.28 (m, 2H), 5.20–5.05 (m, 2.5H), 4.01–3.87 (m, 4H). ESI-MS *m/z* calcd. for C_19_H_20_N_3_O_4_S [M+H] 386.1. Found: 386.0. HRMS for C_19_H_20_N_3_O_4_S [M+H]^+^ 386.1169. Found: 386.1169.

### 3.3. Fungicidal Assays

Each of the test compounds was dissolved in DMSO (10 mL). Fungicidal activities of compounds **F**, **G**, **H**, **I** and **J** against *Sclerotinia sclerotiorum* *(Lib.)* de Bary, *Phytophthora parasitica* Dast, *Botrytis cinerea Pers.*, *Rhizoctonia solani* Kühn., *Pyricularia oryzae Cav.* and *Phoma asparagi Saecrdo* were evaluated using the mycelium growth rate test as previously reported [40].

Inhibition rates of compounds **F** and **G** against *S. sclerotiorum*, *P. Parasitica* Dast, *B. cinerea*, *R solani*, *P. oryzae* Cav and *P. asparagi Saecrdo* at 50 μg/mL were determined first and the results are given in Table 2. The inhibition rate of compounds **F**, **G**, **H**, **I** and **J** against *S. sclerotiorum* were further determined at the concentrations of 50, 20, 10, 5 and 2 μg/mL, respectively. Then EC_50_ and EC_90_ values were estimated using logit analysis [41]. The commercial fungicide chlorothalonil was used as a control in the above bioassay.

### 3.4. Enzyme Inhibitory Activities Bioassay

Inhibitory activities of all the synthesized compounds towards *Candida albicans* GlcN-6-P synthase were evaluated using the mycelium growth rate test as previously reported [38]. Three replicates were performed. Absorbance at λ = 585 nm was measured and GlcN-6-P concentration in the sample was read from the standard curve (solutions of glucosamine-HCl (0.1–1 mM) were assayed simultaneously, to obtain a standard line from the plot of extinction against concentration of glucosamine). In each experiment, two control samples, one without enzyme and one without substrates, were assayed in the same way.

## 4. Conclusions

In summary, a series of novel glycosylthiadiazole derivatives were synthesized, and their bioactivities were evaluated. The bioassays showed that they had the inhibitory activities against glucosamine-6-phosphate synthase, and at the same time, the results from the Chinese Academy of Agricultural Science have shown that most of the tested compounds have good fungicidal activities against *S. sclerotiorum.* Among all the novel compounds tested, compounds **F**-**5** and **G**-**8** displayed better fungicidal activities than the commercial fungicide chlorothalonil. The SAR of the designed compounds was studied: compounds with two electron-withdrawing substituents in the benzene ring have better fungicidal activities than those with two electron-donating substituents. The compounds with the protecting groups in the sugar ring have less inhibitory activities against Glms than those without protecting groups, but displayed better fungicidal activities against *S. sclerotiorum*, and the advantage depends on the number and type of the protection groups. Further studies are in progress.

## Supplementary Materials

Supplementary materials can be accessed at: http://www.mdpi.com/1420-3049/19/6/7832/s1.
