# Supplementary material for: Design, Synthesis and Bioactivity of Novel Glycosylthiadiazole Derivatives
Source: Molecules. 2014 Jun 11;19(6):7832–49. doi: 10.3390/molecules19067832 (PMC6271630; doi:10.3390/molecules19067832)

Supporting Information

Figure S1. ^{13}C-NMR spectrum of compound **F-1**.

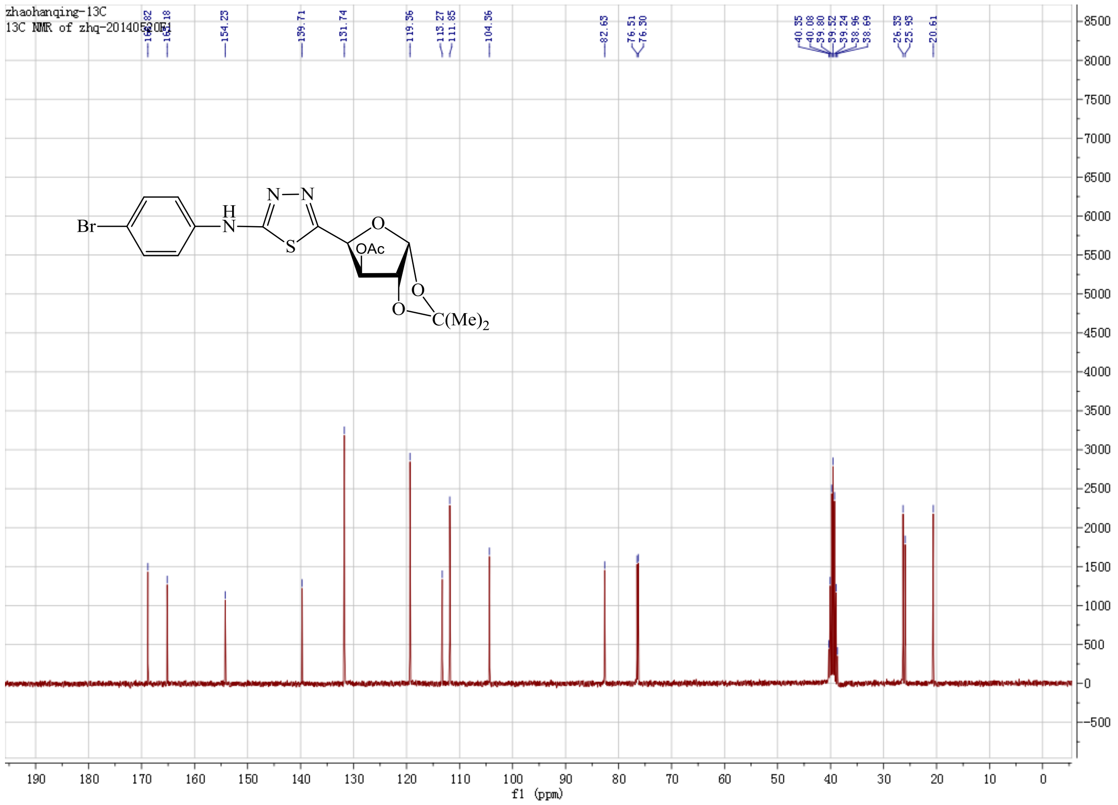

Figure S2. ^{13}C-NMR spectrum of compound **F-2**.

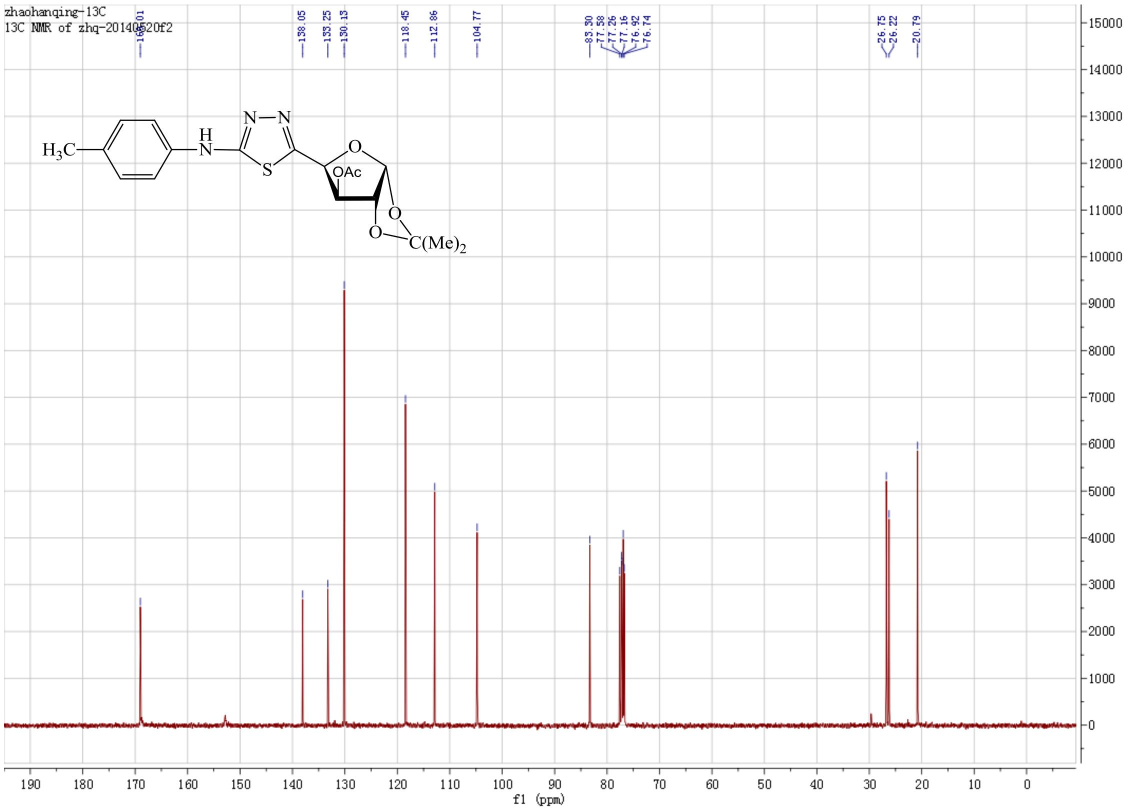

Figure S3. ^{13}C-NMR spectrum of compound **F-3**.

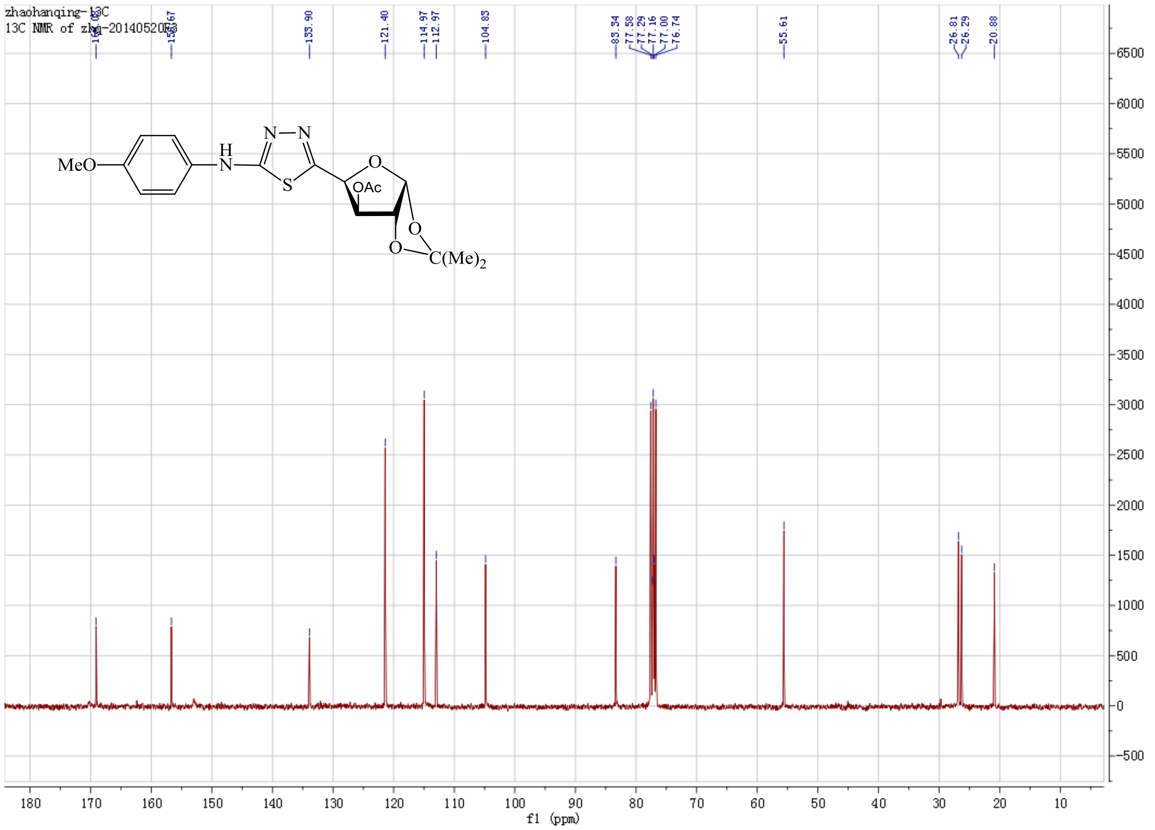

Figure S4. ^{13}C-NMR spectrum of compound **F-4**.

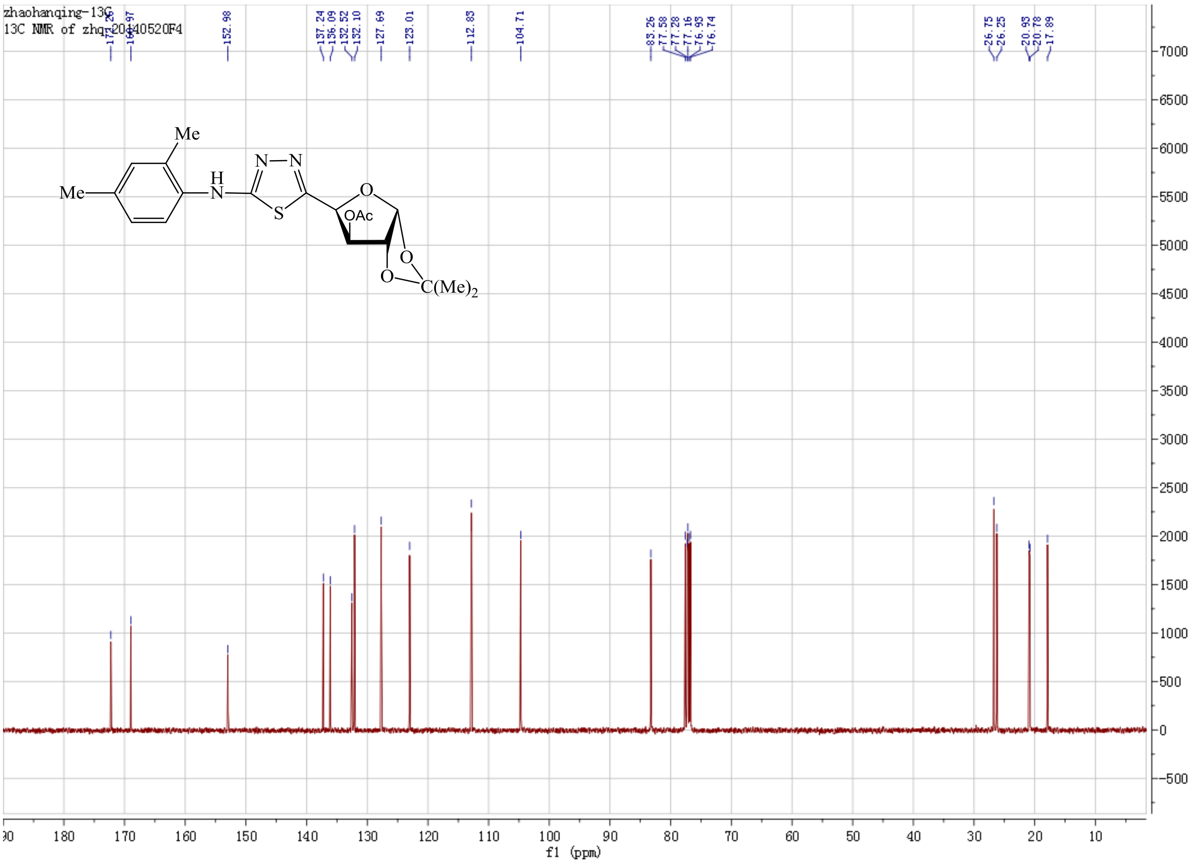

Figure S5. ^{13}C-NMR spectrum of compound **F-5**.

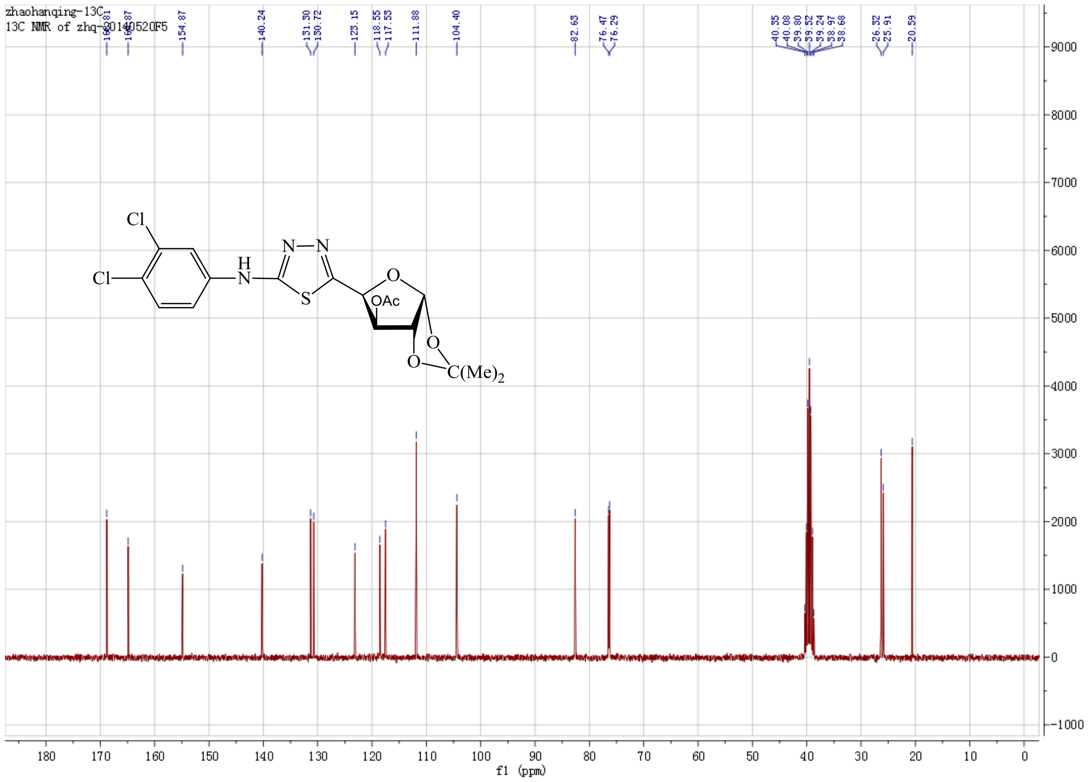

Figure S6. ^{13}C-NMR spectrum of compound **F-6**.

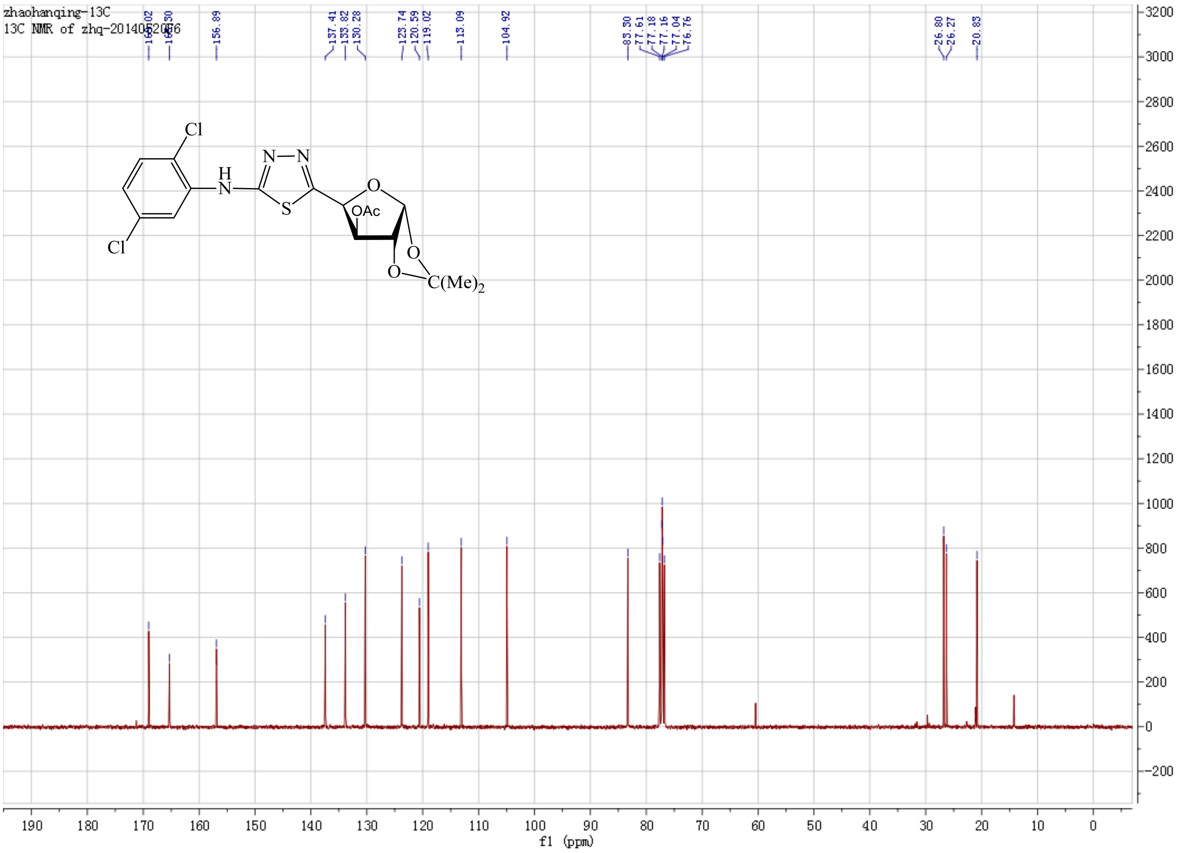

Figure S7. ^{13}C-NMR spectrum of compound **F-7**.

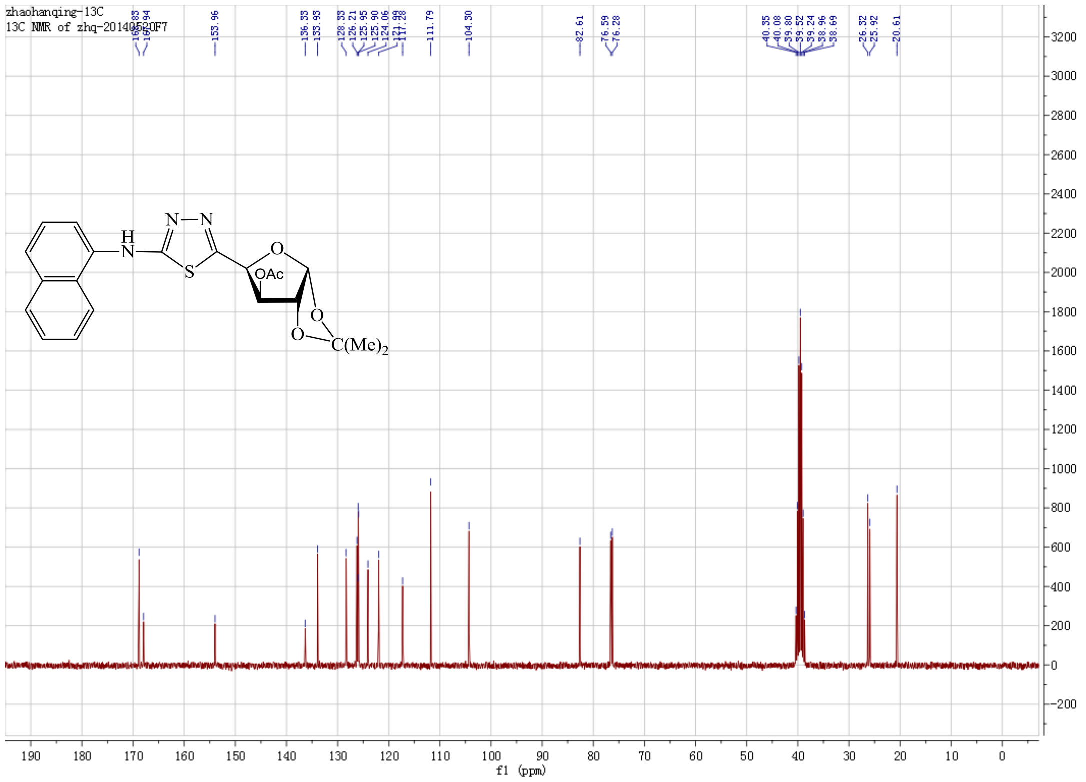

Figure S8. ^{13}C-NMR spectrum of compound **F-8**.

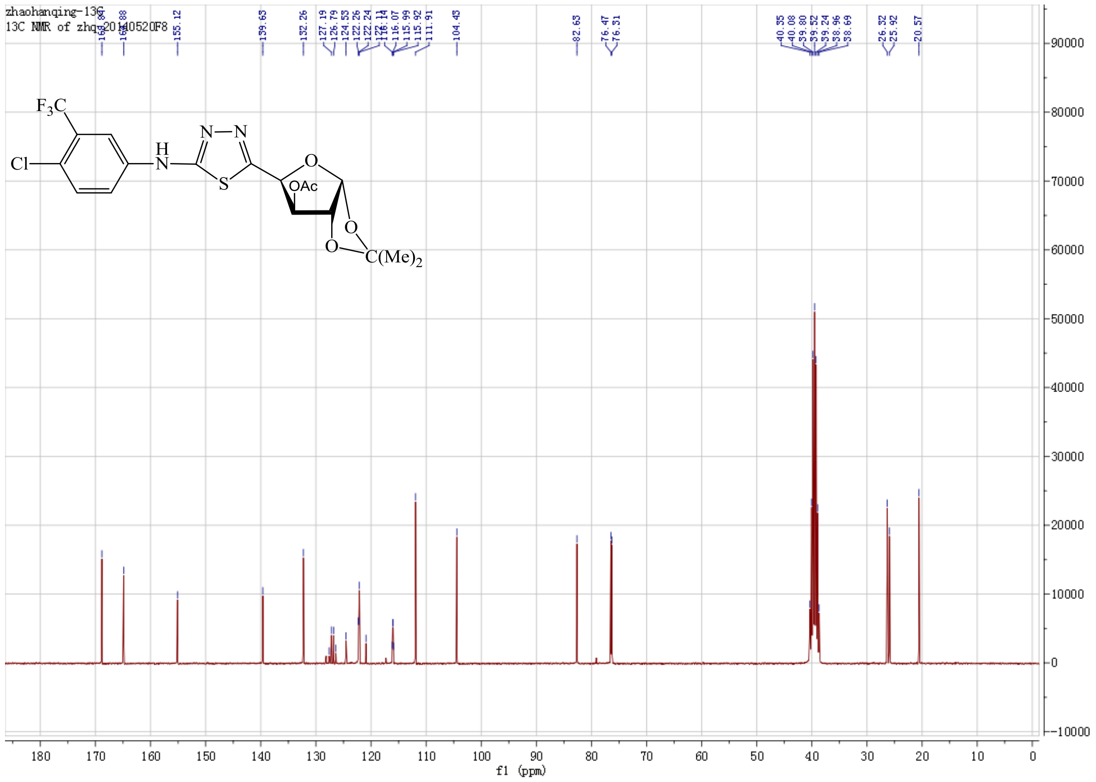

Figure S9. ^{13}C-NMR spectrum of compound **F-9**.

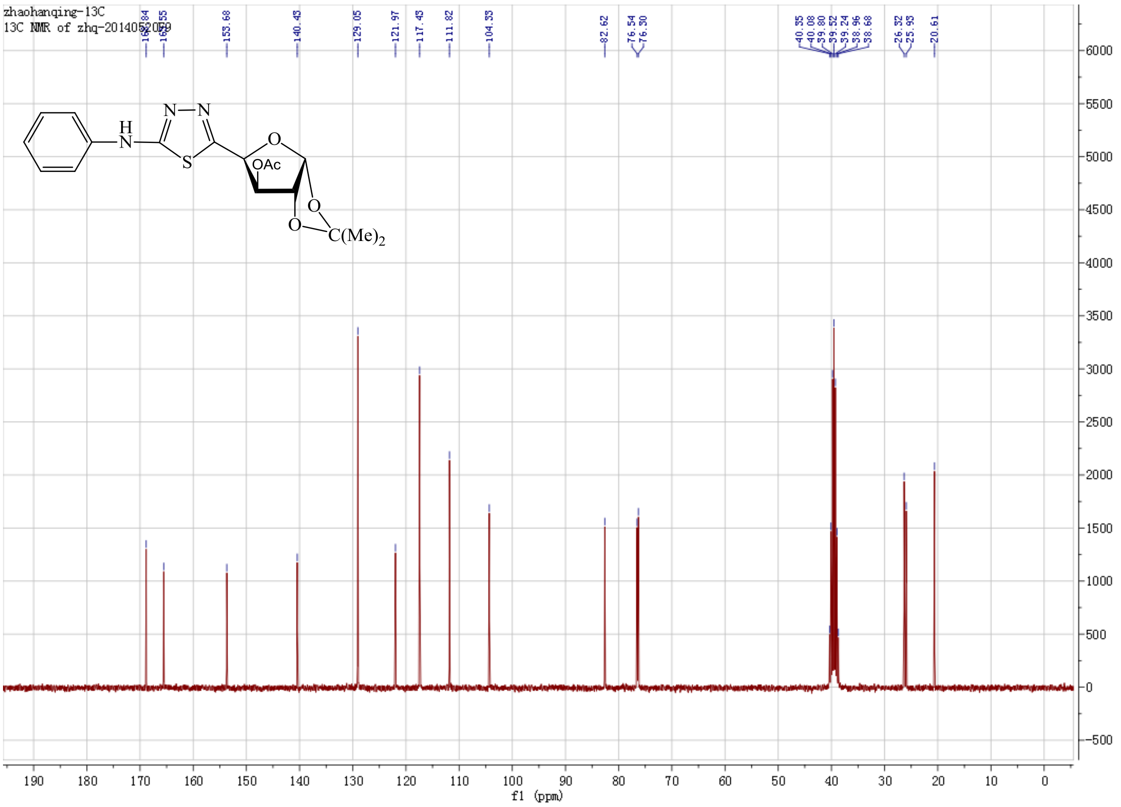

Figure S10. ^{13}C-NMR spectrum of compound **F-10**.

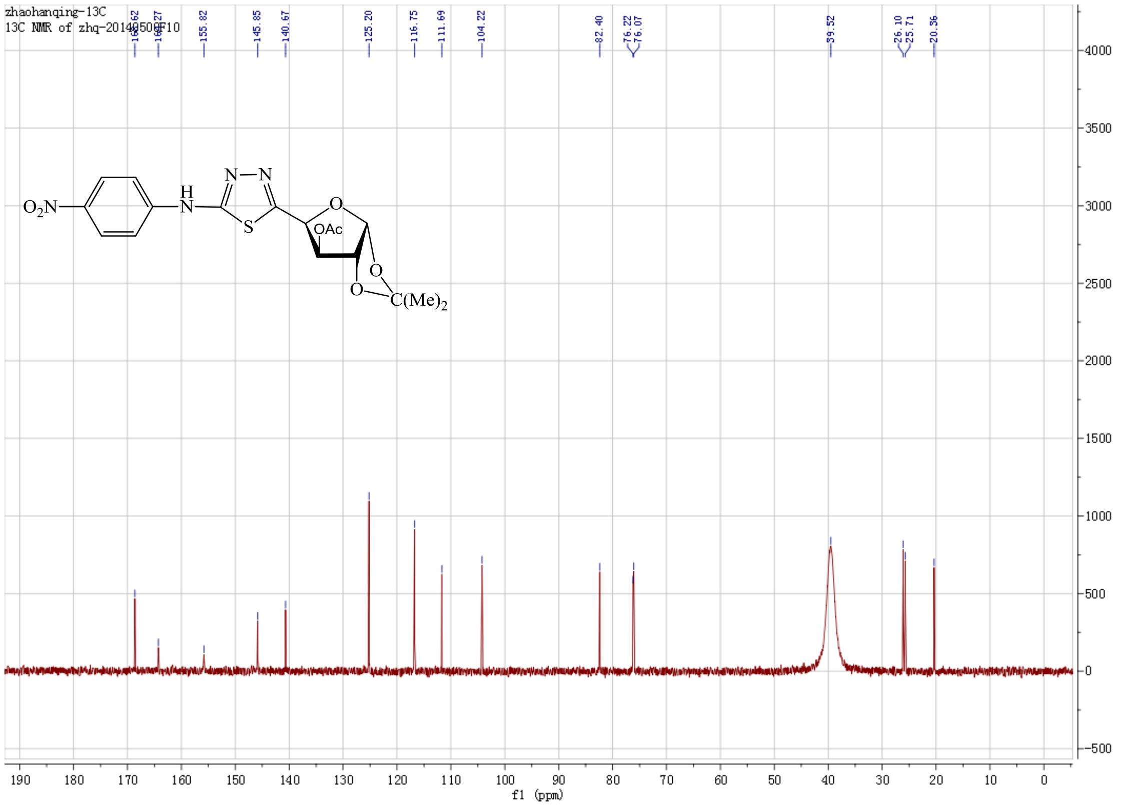

Figure S11. ^{13}C-NMR spectrum of compound **G-1**.

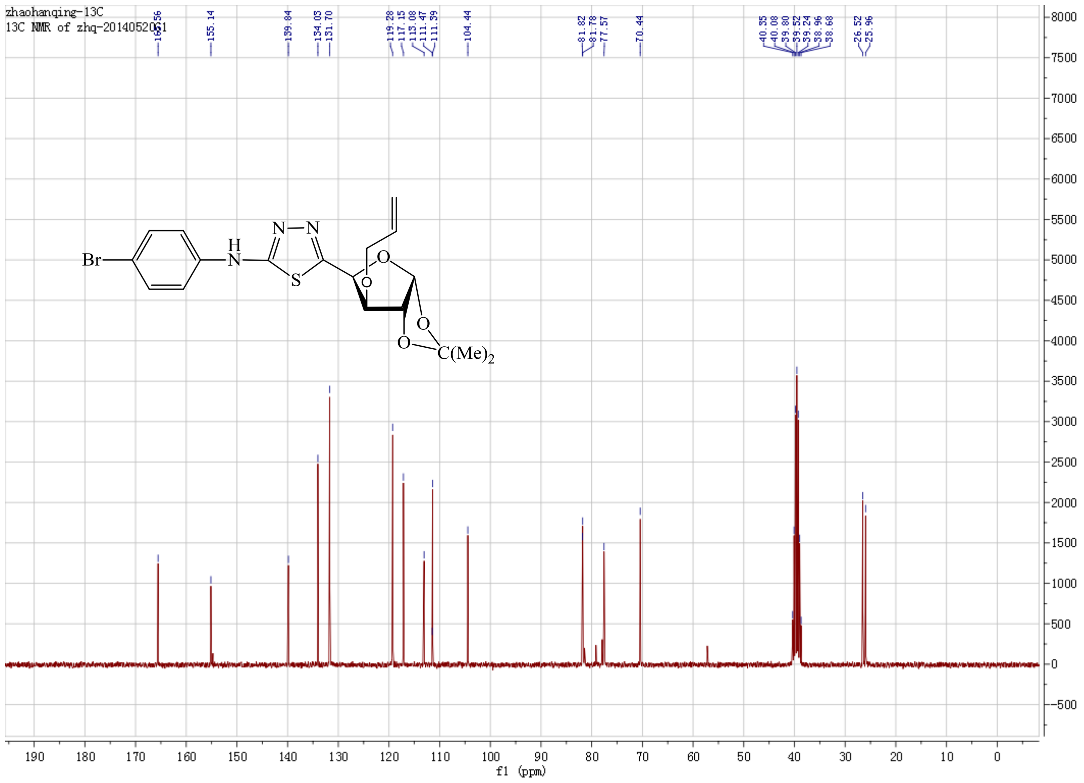

Figure S12. ^{13}C-NMR spectrum of compound **G-2.**

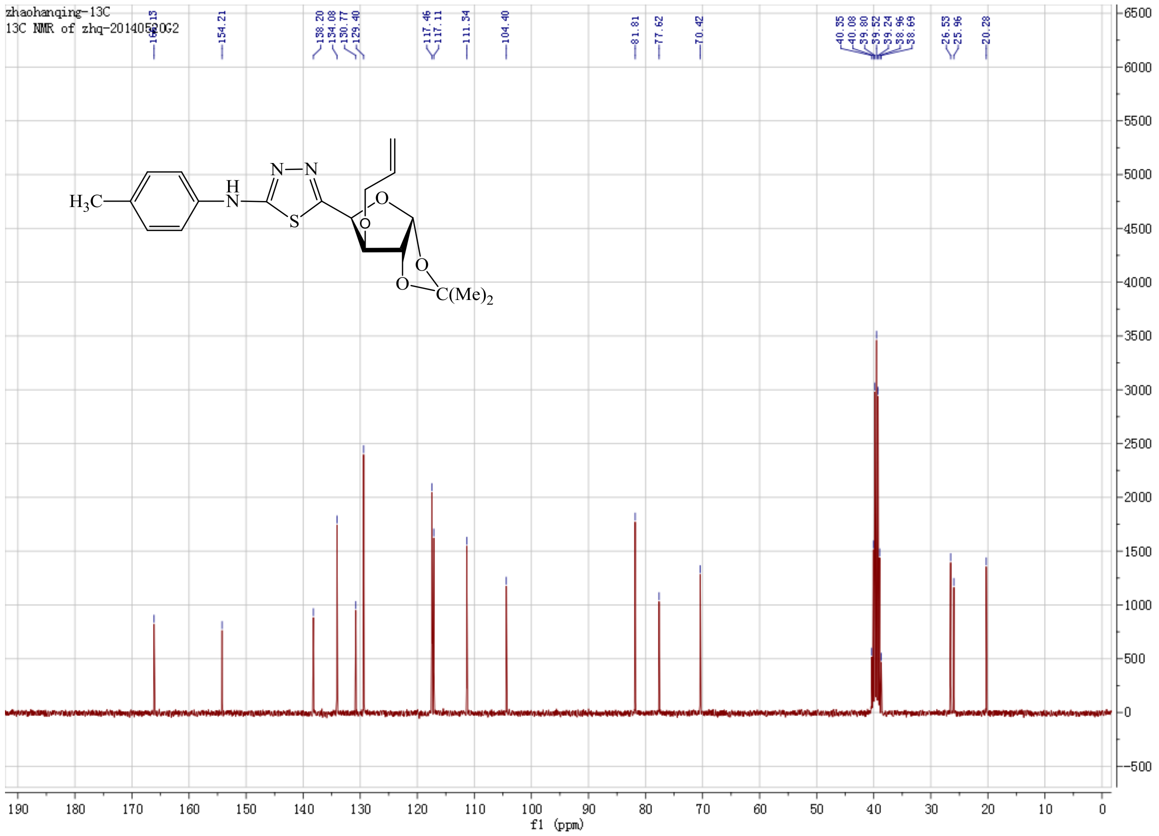

Figure S13. ^{13}C-NMR spectrum of compound **G-3**.

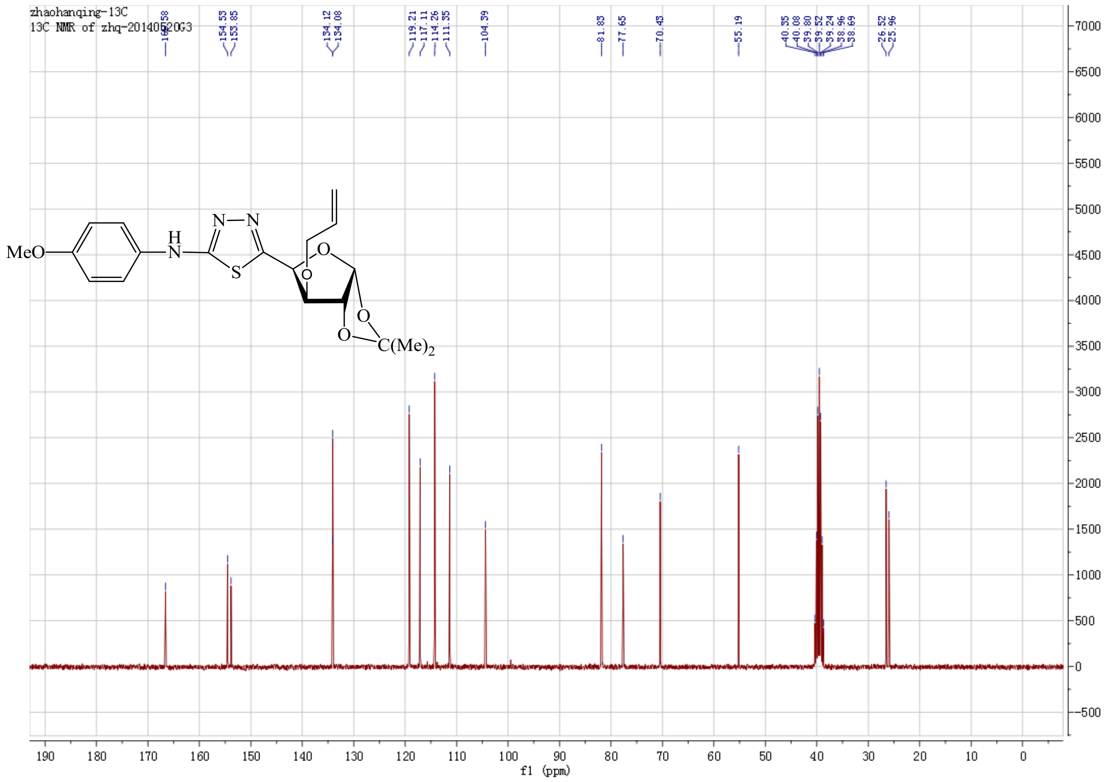

Figure S14. ^{13}C NMR spectrum of compound **G-4**.

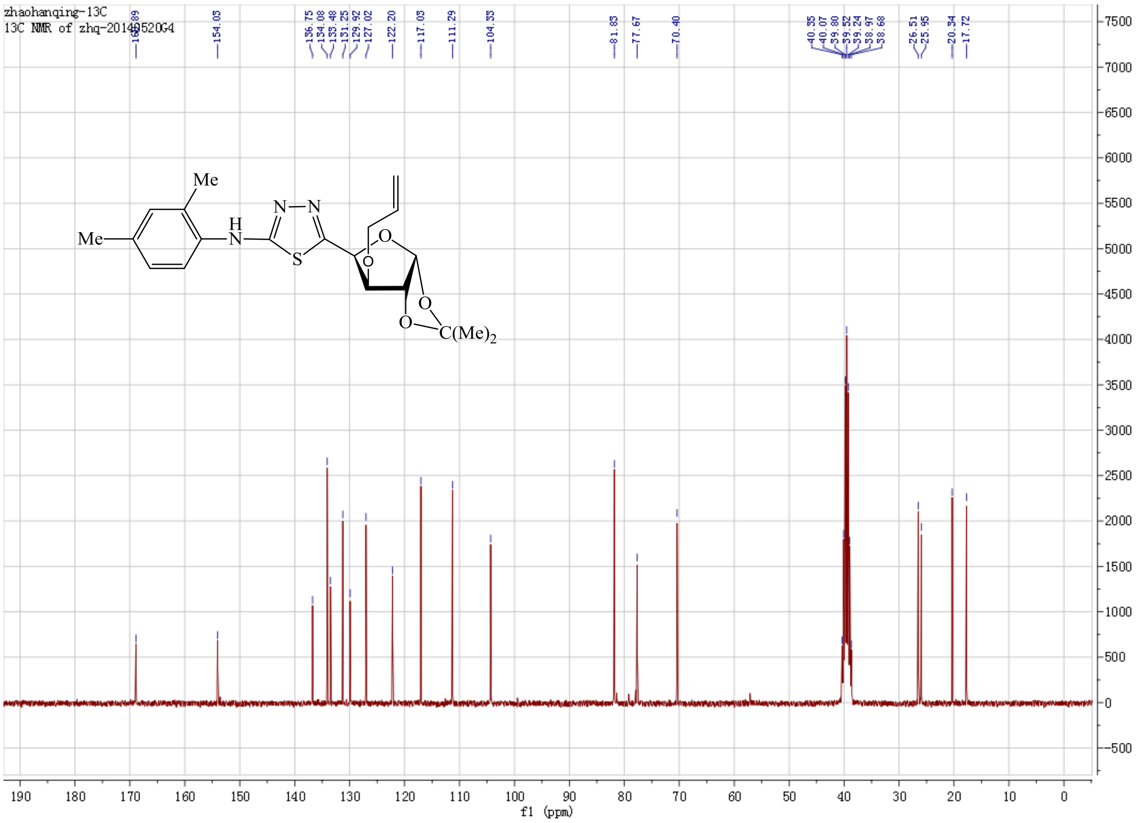

Figure S15. ^{13}C-NMR spectrum of compound **G-5**.

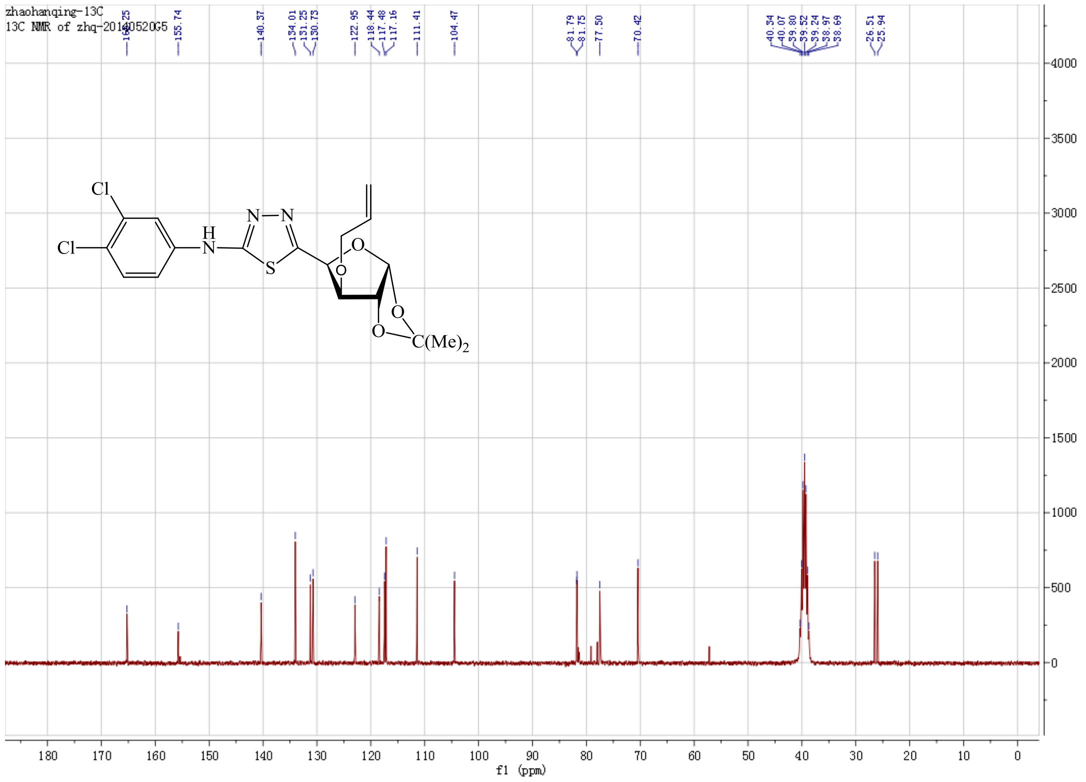

Figure S16. ^{13}C-NMR spectrum of compound **G-6**.

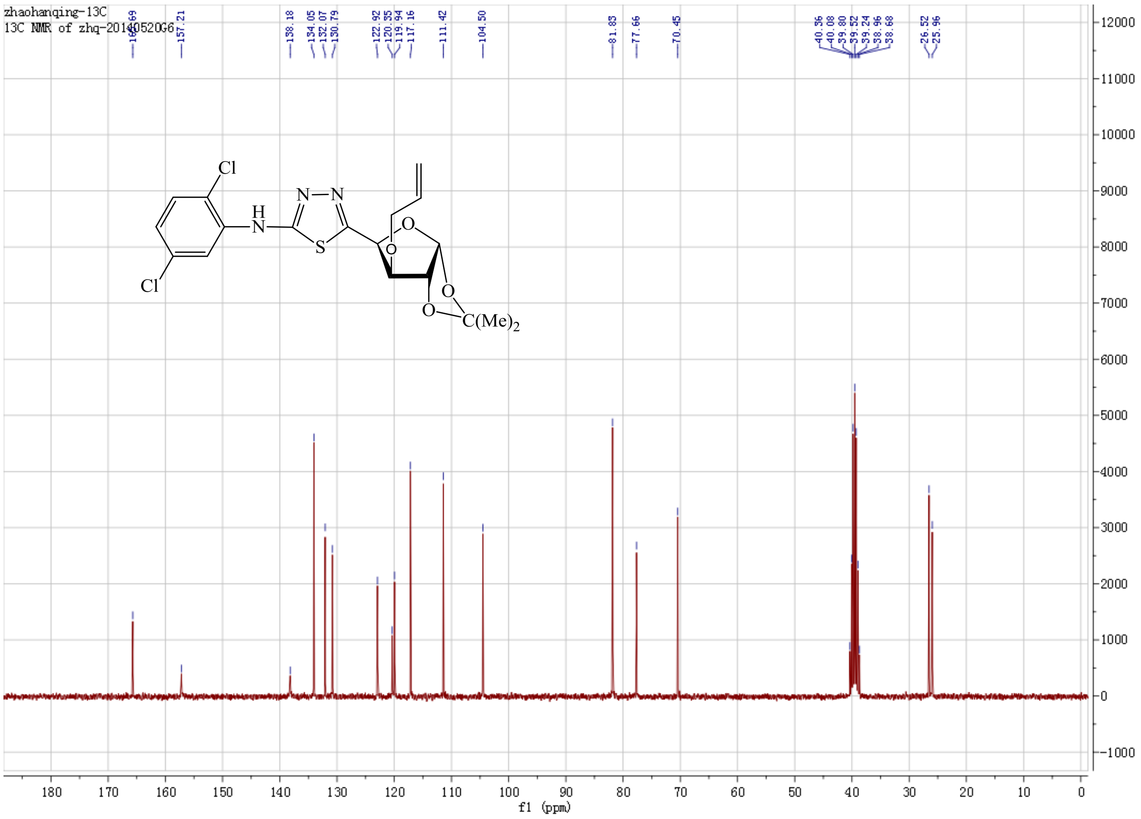

Figure S17. ^{13}C-NMR spectrum of compound **G-7**.

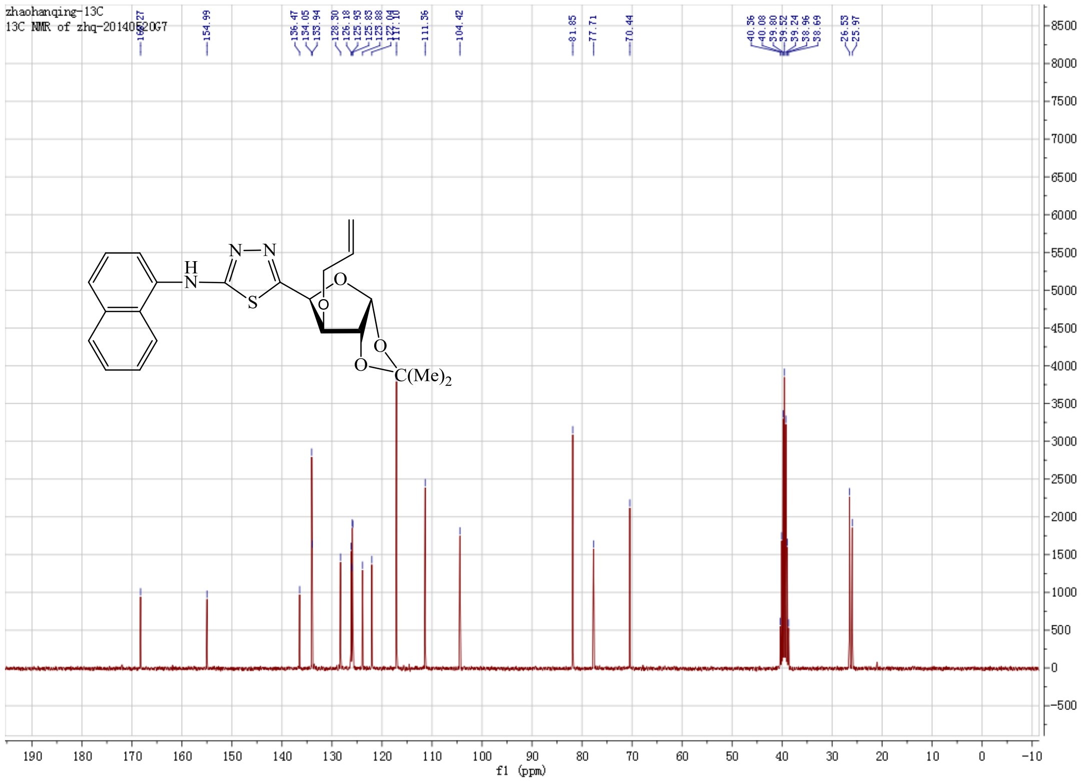

Figure S18. ^{13}C-NMR spectrum of compound **G-8**.

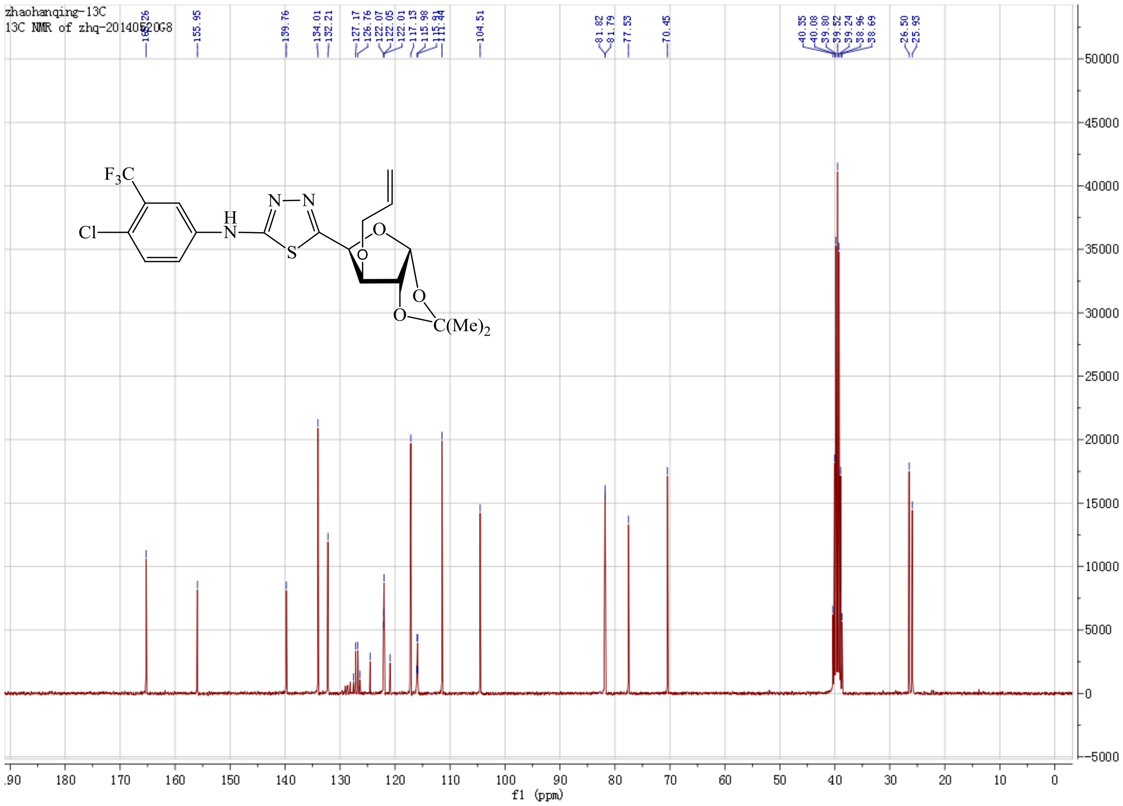

Figure S19. ^{13}C-NMR spectrum of compound **G-9**.

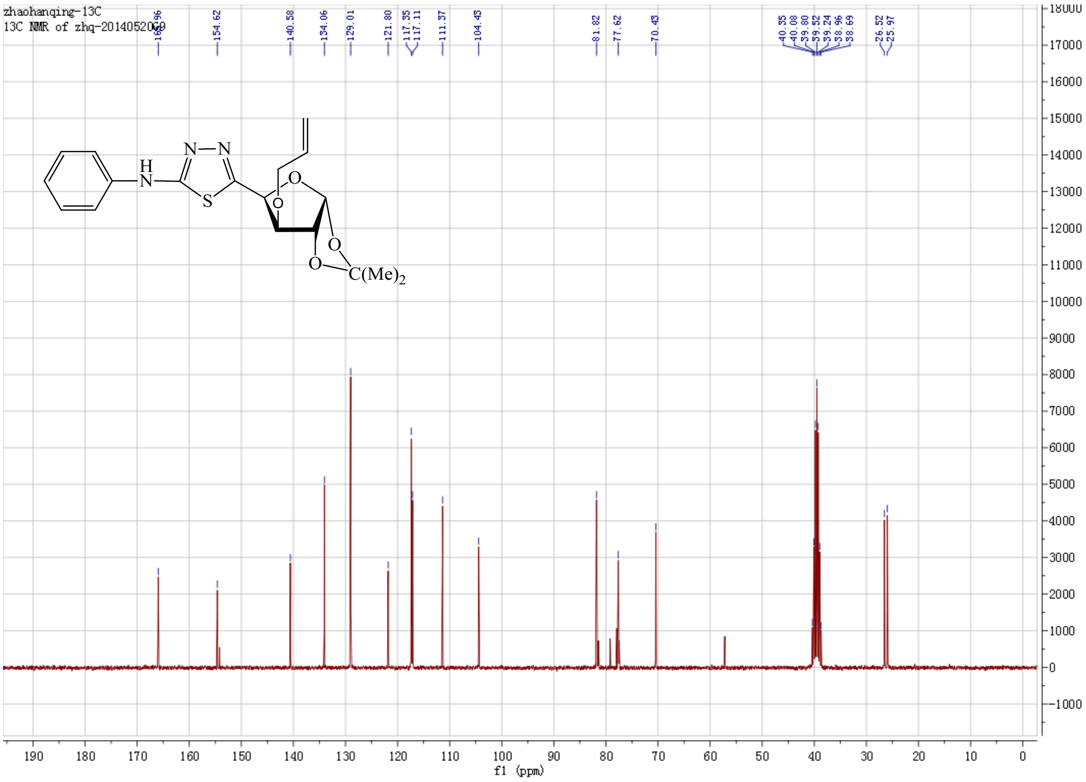

Figure S20. ^{13}C-NMR spectrum of compound **G-10**.

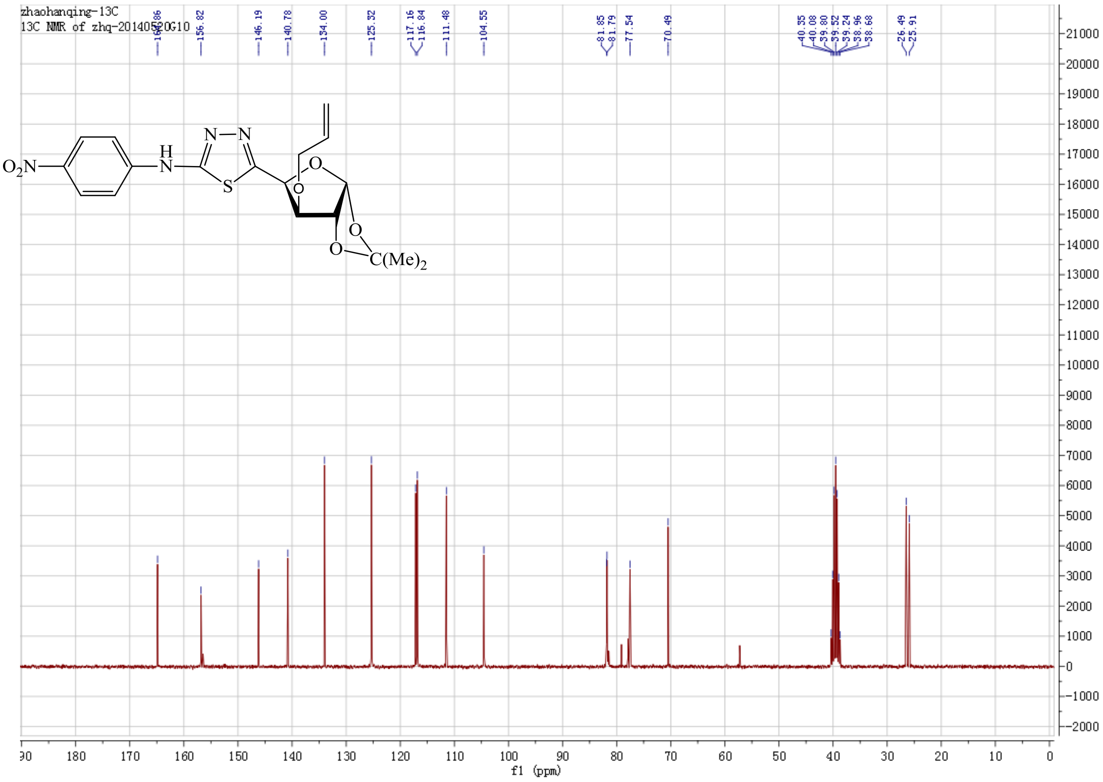

Figure S21. ^{13}C-NMR spectrum of compound **H**.

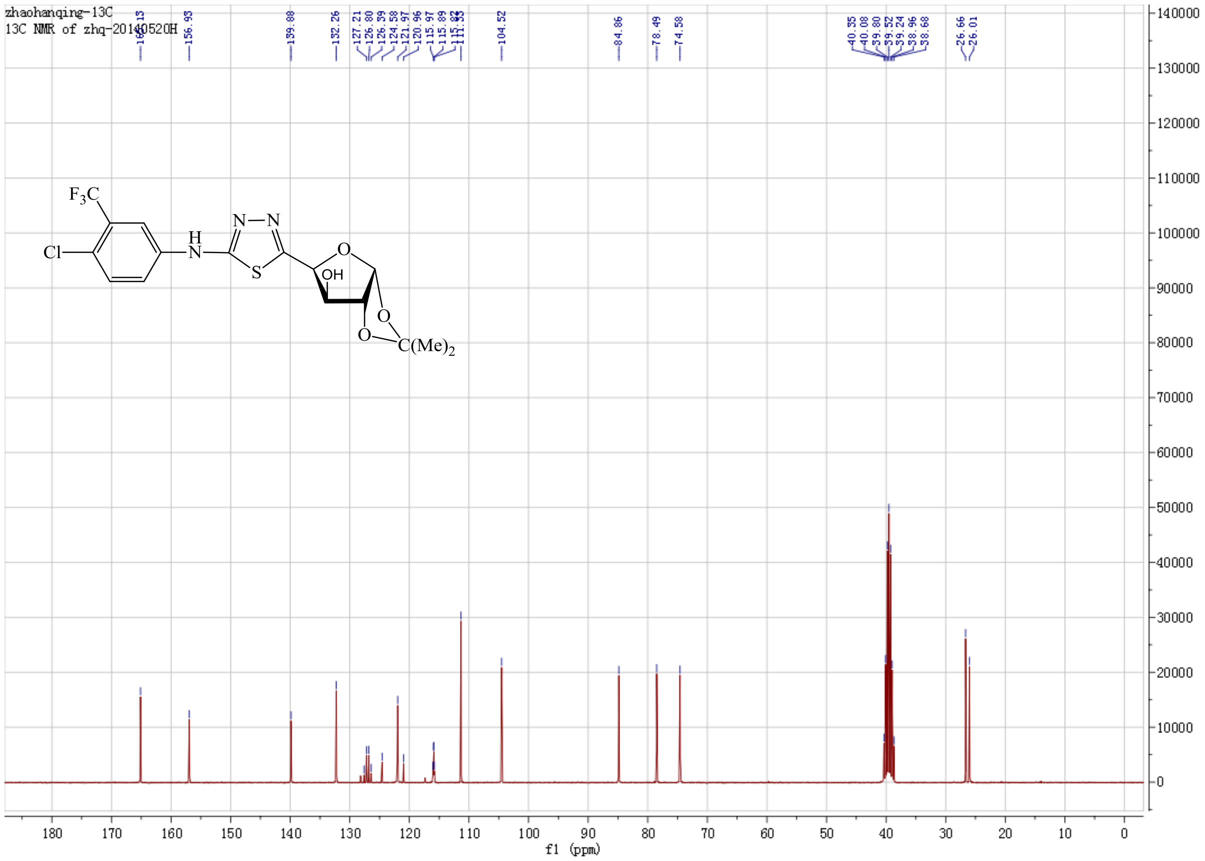

Supplement: Supplementary file 1 [file molecules-19-07832-s001.pdf]
